# Structure–Function Engineering of Hydrogel–MOF Polymer Composites for Regenerative Wound Dressings with Emerging Antiviral Biointerface Functions

**DOI:** 10.3390/gels12080661

**Published:** 2026-07-23

**Authors:** Irving A. González-Lara, Nallely G. Hernández-Hernández, Lesly K. Usme-Duque, Lía A. Martínez-Berlanga, Grecia D. Ortíz-Hernández, María I. León-Campos, Bertha Puente-Urbina, Miguel A. Medina-Morales, Elan I. Loredo-Alcalá, Leopoldo J. Ríos-González, Thelma K. Morales-Martínez, Roberto Arredondo-Valdés, Adolfo Romero-Galarza, Lucía F. Cano-Salazar, Rebeca Betancourt-Galindo, María O. González-Díaz, Nayeli Rodríguez-Fuentes, Javier Enríquez-Medrano, Florentino Soriano-Corral, Raul Rosales-Ibáñez, Amairany Rodríguez-Navarrete, Denis A. Cabrera-Munguía, Jesús A. Claudio-Rizo

**Affiliations:** 1 Facultad de Ciencias Químicas, Universidad Autónoma de Coahuila, Ing. J. Cárdenas Valdez S/N, República, Saltillo 25280, Mexico; irving.gonzalez@uadec.edu.mx (I.A.G.-L.); nallelyhernandezher@uadec.edu.mx (N.G.H.-H.); katleya_usme@uadec.edu.mx (L.K.U.-D.); lia.berlanga@uadec.edu.mx (L.A.M.-B.); grecia.ortiz99@gmail.com (G.D.O.-H.); ileanaleon@uadec.edu.mx (M.I.L.-C.); miguel.medina@uadec.edu.mx (M.A.M.-M.); leopoldo.rios@uadec.edu.mx (L.J.R.-G.); thelma_morales@uadec.edu.mx (T.K.M.-M.); r-arredondo@uadec.edu.mx (R.A.-V.); a_romero@uadec.edu.mx (A.R.-G.); lucia.cano@uadec.edu.mx (L.F.C.-S.); 2 Departamento de Horticultura, Universidad Autónoma Agraria Antonio Narro, Calz. Antonio Narro 1923, Buenavista, Saltillo 25315, Mexico; 3 Centro de Investigación en Química Aplicada (CIQA), Blvd. Enrique Reyna Hermosillo 140, San José de los Cerritos, Saltillo 25294, Mexico; bertha.puente@ciqa.edu.mx (B.P.-U.); rebeca.betancourt@ciqa.edu.mx (R.B.-G.); javier.enriquez@ciqa.edu.mx (J.E.-M.); florentino.soriano@ciqa.edu.mx (F.S.-C.); 4 Center for Biodiversity Conservation and Ecology of Coahuila, Universidad Autónoma de Coahuila, Miguel Hidalgo 212, Zona Centro, Cuatro Ciénegas 27640, Mexico; elan_laredo@uadec.edu.mx; 5 Centro de Investigación Científica de Yucatán (CICY), Calle 43 No. 130 × 32 y 34, Chuburná de Hidalgo, Mérida 97205, Mexico; maria.gonzalez@cicy.mx (M.O.G.-D.); nayeli.rodriguez@cicy.mx (N.R.-F.); 6 Ministry of Science, Humanities, Technology and Innovation (SECIHTI), Av. Insurgentes Sur 1582, Crédito Constructor, Benito Juárez, Mexico City 03940, Mexico; 7 Tissue Engineering Laboratory and Translational Dentistry Clinic, Facultad de Estudios Superiores Iztacala, National Autonomous University of Mexico (UNAM), Tenayuca-Chalmita S/N, Cuautepec Barrio Bajo, Gustavo A. Madero, Mexico City 07239, Mexico; rosales_ibanez@unam.mx (R.R.-I.); amairany.rodriguez@iztacala.unam.mx (A.R.-N.)

**Keywords:** hydrogel–MOF composites, chronic wound healing, antiviral biointerfaces, structure–function relationships, polymeric wound dressings, biointerface engineering

## Abstract

Chronic wounds constitute a major clinical and socioeconomic burden owing to prolonged inflammation, persistent bacterial infection, impaired angiogenesis, and defective extracellular matrix remodeling. Advanced wound dressings have traditionally been developed to promote tissue regeneration, control bacterial infection, and restore the wound microenvironment. Recent advances have focused on multifunctional biomaterials integrating regenerative, antibacterial, anti-inflammatory, antioxidant, and controlled drug-delivery properties. Within this context, antiviral biointerface engineering has emerged as a promising, although still exploratory, materials-engineering perspective rather than an established function of wound dressings. Hydrogel–metal–organic framework (MOF) hybrid polymer composites have emerged as versatile platforms for multifunctional wound dressings. Hydrogels provide hydrated three-dimensional matrices with tunable porosity, swelling behavior, mechanical compliance, and biocompatibility, whereas MOFs contribute high surface area, adjustable pore architectures, chemically tailorable active sites, and controlled ion release. Their integration generates synergistic systems whose performance is governed by structure–function relationships involving polymer crosslinking density, MOF dispersion, pore hierarchy, interfacial adhesion, swelling dynamics, and surface functionalization. Collectively, these parameters regulate mass transport, mechanical stability, therapeutic delivery, and cytocompatibility while potentially influencing virus–material interactions through engineered biointerfaces. Current evidence indicates that direct experimental demonstrations of antiviral performance in hydrogel–MOF wound dressing systems remain limited. Accordingly, antiviral biointerface functions should be regarded as emerging engineering opportunities requiring further experimental validation before clinical translation. This review critically analyzes the structure–function engineering principles governing hydrogel–MOF hybrid systems and examines how established regenerative functions may be integrated with emerging antiviral biointerface concepts. Unlike previous reviews focused primarily on drug delivery, antibacterial activity, or tissue engineering, this review emphasizes the relationships between polymer architecture, MOF chemistry, interfacial design, and transport phenomena while explicitly distinguishing experimentally supported evidence from prospective mechanistic concepts. Particular attention is given to current limitations, translational challenges, and future directions for the rational design of next-generation multifunctional hydrogel–MOF wound dressings.

## 1. Introduction

Chronic wounds represent a persistent global healthcare challenge due to their prolonged healing time, high recurrence rates, and strong association with infection, inflammation, and tissue degeneration [1,2]. Unlike acute lesions, chronic wounds develop a highly dysregulated microenvironment characterized by ischemia, excessive protease activity, oxidative stress, extracellular matrix (ECM) degradation, abnormal pH, and dense microbial biofilms [3,4]. These factors collectively impair re-epithelialization, angiogenesis, granulation tissue formation, and ECM remodeling, thereby establishing a pathological niche that is difficult to reverse [1,2,3,4]. While bacterial colonization and antimicrobial resistance have traditionally dominated the clinical discussion [5], advanced wound dressings are primarily designed to promote tissue regeneration, control bacterial infection, modulate inflammation, and restore the physiological wound microenvironment. Recent advances have increasingly focused on the development of multifunctional biomaterials capable of integrating regenerative, antibacterial, anti-inflammatory, antioxidant, hemostatic, and controlled drug-delivery functions within a single platform [6,7,8,9]. Current reviews consistently identify tissue regeneration and antibacterial performance as the principal objectives of advanced wound dressings, whereas antiviral biointerface functions remain an emerging research direction rather than an established strategy for chronic wound management [10,11]. Consequently, antiviral functionalities should be regarded as complementary design opportunities for next-generation multifunctional wound dressings rather than as responses to a recognized viral pathology in chronic wounds.

Importantly, the present review does not propose that chronic wounds constitute clinically relevant reservoirs of enveloped viruses or that antiviral activity should be considered a current therapeutic requirement in chronic wound management. Instead, it examines whether the physicochemical principles that already govern the rational design of regenerative hydrogel–MOF wound dressings may also provide a scientifically grounded framework for exploring future antiviral biointerface functions as an emerging biomaterials engineering strategy.

This emerging perspective highlights the need for multifunctional wound dressings that move beyond passive coverage or solely antibacterial action [12,13]. Advanced materials for chronic wound care must simultaneously regulate moisture, absorb exudate, modulate inflammation, support cell migration, and reduce the burden of diverse pathogens [14]. Among available biomaterials, hydrogels have attracted substantial attention because their highly hydrated three-dimensional polymeric networks closely resemble native soft tissues and can provide an optimal healing microenvironment [15,16]. Natural and synthetic hydrogel systems offer tunable swelling behavior, porosity, degradation kinetics, and mechanical compliance, enabling tailored control over oxygen permeability, nutrient transport, and therapeutic release [15,16]. Furthermore, by adjusting crosslinking density, polymer composition, charge distribution, and mesh architecture, hydrogels can be engineered as active matrices for selective retention of biomolecules, nanoparticles (NPs), or infectious particles while preserving cytocompatibility [15,16,17].

Despite these advantages, conventional hydrogels often display limited intrinsic antiviral functionality. Their regenerative benefits alone may not be sufficient to incorporate emerging antiviral biointerface functions into multifunctional wound dressing platforms. In this context, MOFs have emerged as a highly versatile class of porous crystalline materials with unique relevance for biomedical interfaces [18,19]. Constructed from metal nodes and organic linkers, MOFs exhibit exceptionally high surface area, tunable pore dimensions, controllable surface chemistry, and accessible functional sites [18,19]. These characteristics enable interactions with viral envelopes, capsid proteins, and associated biomacromolecules through electrostatic attraction, coordination bonding, hydrophobic partitioning, hydrogen bonding, and steric confinement [20,21]. In addition, selected MOFs containing Zn, Cu, Ag, Ti, Fe, or Zr may contribute antiviral activity through controlled ion release, catalytic generation of reactive oxygen species (ROS), or disruption of protein structure [18,19,22].

However, pristine MOFs alone are often brittle, difficult to process, and suboptimal for direct application onto dynamic wound beds [23]. Their integration within hydrogel matrices therefore represents a rational materials-engineering strategy to merge the complementary advantages of both platforms [24,25]. Hydrogel–MOF hybrid polymer composites combine the softness, moisture retention, and tissue compatibility of polymer networks with the adsorption capacity, structural porosity, and chemical functionality of MOFs. More importantly, their performance is not simply additive, but governed by multiscale structure–function relationships involving MOF particle dispersion, interfacial adhesion, hydrogel crosslink density, hierarchical pore organization, swelling dynamics, surface charge, and ligand functionalization [24,25,26].

Such structure–function relationships are expected to influence the interaction of hydrogel–MOF composites with biological molecules and microorganisms and provide a rational basis for exploring emerging antiviral biointerface functions. Recent studies suggest that interactions with enveloped viruses may arise through complementary physicochemical mechanisms, including electrostatic attraction, hydrogen bonding, multivalent interactions, diffusion within hydrated polymeric matrices, and non-covalent surface adsorption. However, direct experimental evidence demonstrating these mechanisms in hydrogel–MOF wound dressing systems remains limited, highlighting an important opportunity for future research [26,27,28]. Simultaneously, the surrounding hydrogel phase can preserve a moist and regenerative microenvironment favorable for fibroblast proliferation, keratinocyte migration, and tissue remodeling [24,25,26,29,30]. This dual functionality introduces a central design challenge: maximizing pathogen control without compromising cellular compatibility or delaying wound closure.

**Figure 1:**
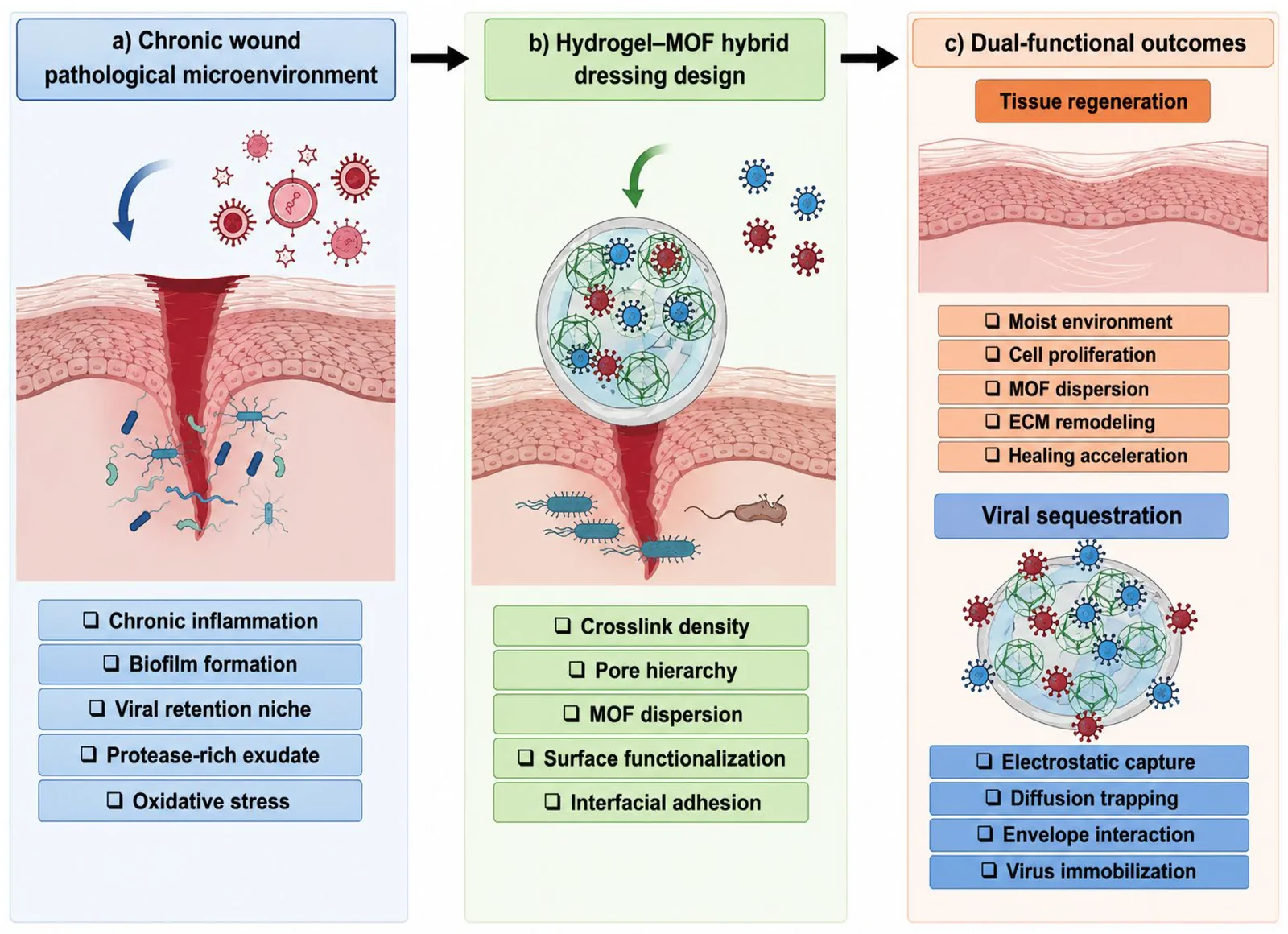
Structure–function concept of hydrogel–MOF hybrid dressings for chronic wound regeneration and enveloped virus sequestration. Created in BioRender. Rizo, J. (2026) https://BioRender.com/73e680q (accessed on 20 July 2026).

Accordingly, hydrogel–MOF composites are emerging as next-generation smart dressings for complex wound care [24,25,26,29,30,31,32]. Unlike previous reviews on hydrogel–MOF composites, which have primarily focused on antibacterial activity, drug delivery, tissue engineering, or general biomedical applications, the present review critically discusses how rational structure–function engineering principles may guide the integration of established regenerative functions with emerging antiviral biointerface concepts. Particular emphasis is placed on distinguishing experimentally demonstrated evidence from future perspectives, identifying current limitations, and discussing the translational challenges associated with incorporating antiviral biointerface functions into clinically relevant wound dressing technologies. This review examines the structure–function engineering principles that govern these hybrid materials, with emphasis on how polymer architecture, MOF chemistry, interfacial design, and transport phenomena can be tailored to achieve multifunctional regenerative performance while exploring emerging antiviral biointerface functions (Figure 1). By integrating concepts from polymer science, porous materials engineering, and biointerface design, this review provides a structure–function framework for the rational development of next-generation hydrogel–MOF wound dressings while identifying current evidence, existing knowledge gaps, and future translational opportunities.

## 2. Physicochemical Characteristics of Chronic Wounds and Implications for Multifunctional Biomaterial Design

### 2.1. Clinical Relevance and Microenvironmental Complexity of Chronic Wounds

Chronic wounds represent complex pathological microecosystems that remain a major global health challenge due to their impaired healing dynamics and persistent inflammatory state. These environments are governed by interconnected factors, including ischemia, chronic inflammation, oxidative stress, ECM degradation, and the formation of polymicrobial biofilms. Collectively, these processes disrupt granulation tissue formation, angiogenesis, and ECM remodeling, ultimately leading to delayed or non-healing lesions [33,34,35,36,37]. Beyond their biological complexity, these pathological features provide important design considerations for the development of advanced multifunctional biomaterials aimed at restoring tissue homeostasis and improving wound healing outcomes [8,9].

### 2.2. Physicochemical Characteristics of the Chronic Wound Microenvironment and Implications for Multifunctional Biomaterial Design

Although bacterial infection remains the principal microbiological concern in chronic wounds, the physicochemical characteristics of the wound microenvironment also provide important design considerations for advanced multifunctional biomaterials. The coexistence of protein-rich exudate, degraded ECM, and structured biofilms generates a heterogeneous environment characterized by hydrated polymeric networks that regulate diffusion, molecular transport, and physicochemical interactions at biological interfaces. Rather than considering chronic wounds as established viral retention niches, these physicochemical characteristics may inspire the rational design of hydrogel-based biomaterials with emerging antiviral biointerface functions, although direct evidence supporting antiviral applications in wound dressing systems remains limited [9,26,38,39]. These physicochemical characteristics may influence biomaterial design through coupled mechanisms involving physical confinement within polymeric networks composed of proteins, extracellular DNA (eDNA), and polysaccharides, as well as multivalent electrostatic, hydrophobic, and surface-mediated interactions at biological interfaces [33,34,36,37,40,41].

Importantly, the rationale discussed throughout this review should be interpreted as a biomaterials engineering perspective that draws upon established physicochemical principles of hydrogel and hydrogel–MOF design, rather than as evidence supporting a clinically relevant role of enveloped viruses in chronic wound pathology.

For clarity, throughout this review, the term viral retention is used to describe the physicochemical confinement of viral particles within biological or biomaterial matrices through diffusion restriction or surface interactions. The term viral sequestration is used operationally to describe the intentional capture or immobilization of viral particles by engineered hydrogel–MOF systems through selective physicochemical interactions. In contrast, viral persistence refers to the prolonged maintenance of viral material or infectivity within a biological environment. These terms are used according to these operational definitions throughout this review [7,42].

The structural heterogeneity of the wound bed, including microclots, cellular debris, and fragmented collagen fibers, further restricts molecular diffusion and contributes to the physicochemical complexity of the local wound microenvironment [34,40,43]. Notably, pH plays a central role in modulating these interactions. While healthy skin exhibits mildly acidic conditions (pH 4.1–5.8), chronic wounds shift toward alkaline values (pH 7.4–8.9), enhancing pathogen proliferation and altering the charge of ECM components and biological macromolecules. This promotes adhesion to degraded substrates such as collagen and fibronectin, whose exposure is increased under proteolytic conditions [4].

**Figure 2:**
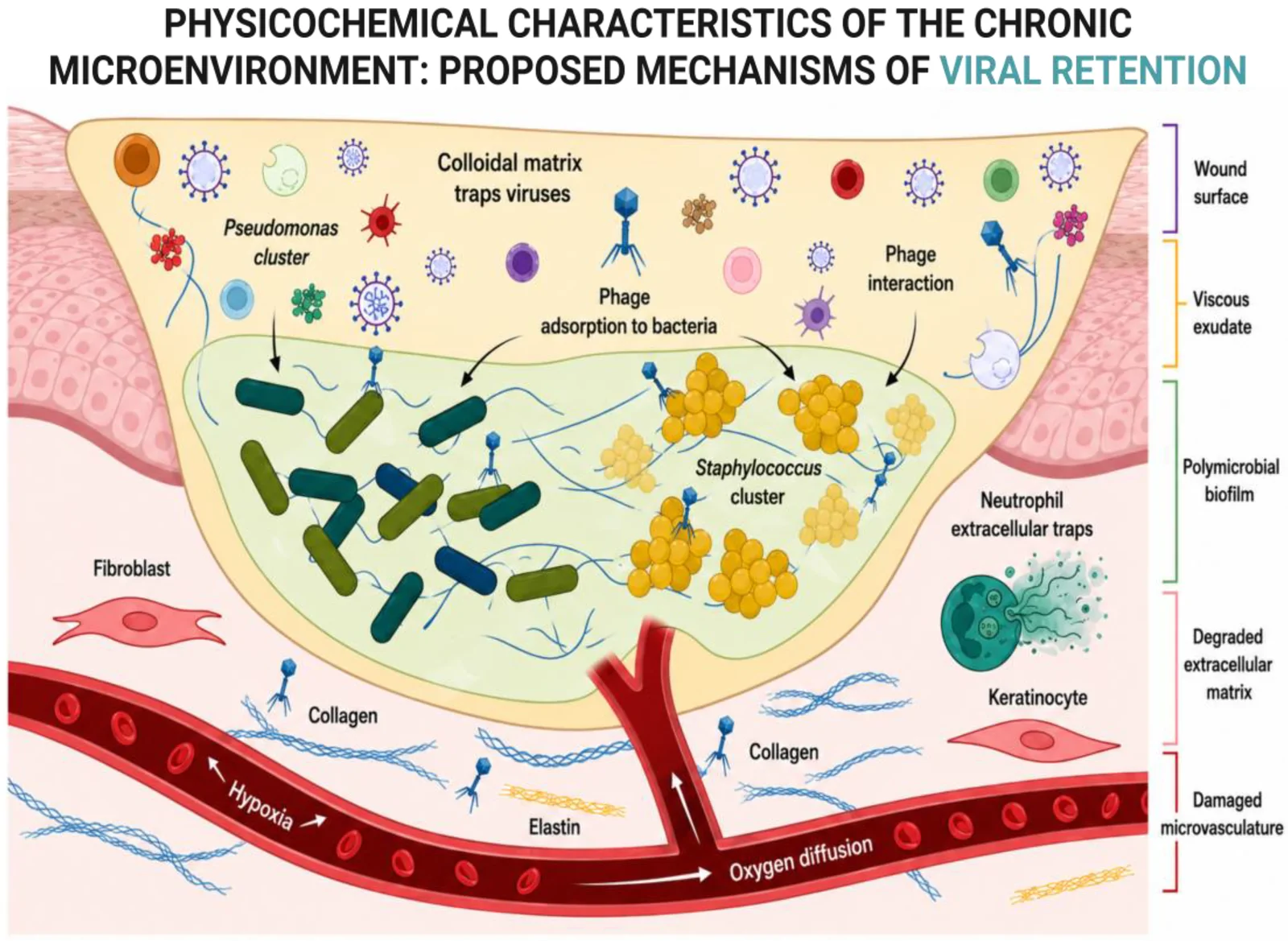
Cross-sectional schematic illustrating the physicochemical characteristics of the chronic wound microenvironment and proposed mechanisms that may contribute to viral retention, including restricted diffusion within protein-rich exudate, interactions with polymicrobial biofilms, and association with degraded ECM components. Created in BioRender. Rizo, J. (2026) https://BioRender.com/67765yn (accessed on 20 July 2026).

Exudate composition also contributes to this physicochemical complexity. Its high content of proteins, cytokines, ROS, and proteases forms a viscous colloidal medium with restricted diffusion, facilitating molecular transport and biointerface interactions within the wound microenvironment [35,36,44]. These physicochemical characteristics are consistent with current strategies for developing multifunctional hydrogel and hydrogel–MOF systems capable of combining moisture management, tissue regeneration, antibacterial activity, and controlled therapeutic delivery. Although antiviral biointerface engineering remains an emerging research direction, these microenvironmental features may also inform the future design of multifunctional biomaterials with potential antiviral biointerface functions (Figure 2) [9,10,11].

### 2.3. Structural and Biological Characteristics of Chronic Wound Biofilms and Implications for Multifunctional Biomaterial Design

Microbial biofilms constitute the dominant structural component of chronic wounds, acting as both protective barriers and complex physicochemical matrices. Their composition—rich in ECM fragments, exopolysaccharides (EPSs), lipids, proteins, and eDNA—limits the efficacy of conventional antimicrobials while generating heterogeneous biological interfaces that regulate molecular transport, diffusion, and interactions with biomaterials [4,38]. These structural and physicochemical characteristics have inspired the development of advanced multifunctional wound dressings designed to improve biofilm management while simultaneously promoting tissue regeneration [9,10,11].

In addition, chronic wounds host complex viral populations, including bacteriophages that interact dynamically with bacterial communities. This coexistence promotes horizontal gene transfer, modulates bacterial virulence, and contributes to antimicrobial tolerance, reinforcing the persistence of polymicrobial ecosystems (Figure 2) [33,45]. Current evidence indicates that bacteriophages constitute the predominant viral component identified in chronic wound ecosystems, whereas evidence supporting a significant role for enveloped viruses remains limited. Consequently, emerging antiviral biointerface strategies should be interpreted as opportunities to expand the functionality of next-generation hydrogel–MOF biomaterials rather than as responses to an established viral pathology in chronic wounds [10,46].

### 2.4. Microenvironmental Dysregulation and Biomaterial-Based Therapeutic Implications

Microenvironmental dysregulation, particularly hypoxia and redox imbalance, plays a central role in chronic wound pathology and has increasingly inspired the rational design of advanced multifunctional biomaterials [47,48,49]. Limited oxygen diffusion promotes biofilm formation, while ROS alter the physicochemical properties of the wound milieu, including hydrophobicity and charge distribution, thereby influencing cellular behavior, ECM remodeling, inflammatory signaling, and biointerface interactions [4,35,36,40,50]. Additionally, immune dysregulation plays a critical role. Dysfunctional neutrophils release neutrophil extracellular traps (NETs), composed of DNA and proteins, which increase local viscosity and create additional polymeric networks that contribute to chronic inflammation and modify the physicochemical characteristics of the wound microenvironment [37,50]. Although NETs are increasingly recognized as important contributors to chronic wound pathology, their application as direct biomaterial design targets remains less established than pH-, ROS-, hypoxia-, biofilm-, or exudate-related cues [51].

Collectively, these findings support the concept that chronic wounds should be regarded as dynamic pathological microenvironments whose physicochemical characteristics provide a rational framework for engineering next-generation multifunctional biomaterials. Advanced hydrogels and hydrogel–MOF composites can be designed to respond to pH imbalance, oxidative stress, excessive exudate, hypoxia, biofilm formation, and inflammatory mediators, thereby integrating regenerative, antibacterial, antioxidant, immunomodulatory, and controlled therapeutic delivery functions [38,52,53,54]. Within this framework, it may be hypothesized that these same physicochemical design principles could potentially be extended to incorporate emerging antiviral biointerface functions in future hydrogel–MOF systems. Although this concept remains largely unexplored and requires experimental validation, it represents a promising direction for expanding the functional capabilities of advanced wound dressings beyond their established regenerative and antibacterial roles (Table 1).

## 3. Polymer Hydrogel Networks as Tunable Diffusion and Retention Matrices

### 3.1. Design Principles of Polymeric Hydrogels for Controlled Transport

**Figure 3:**
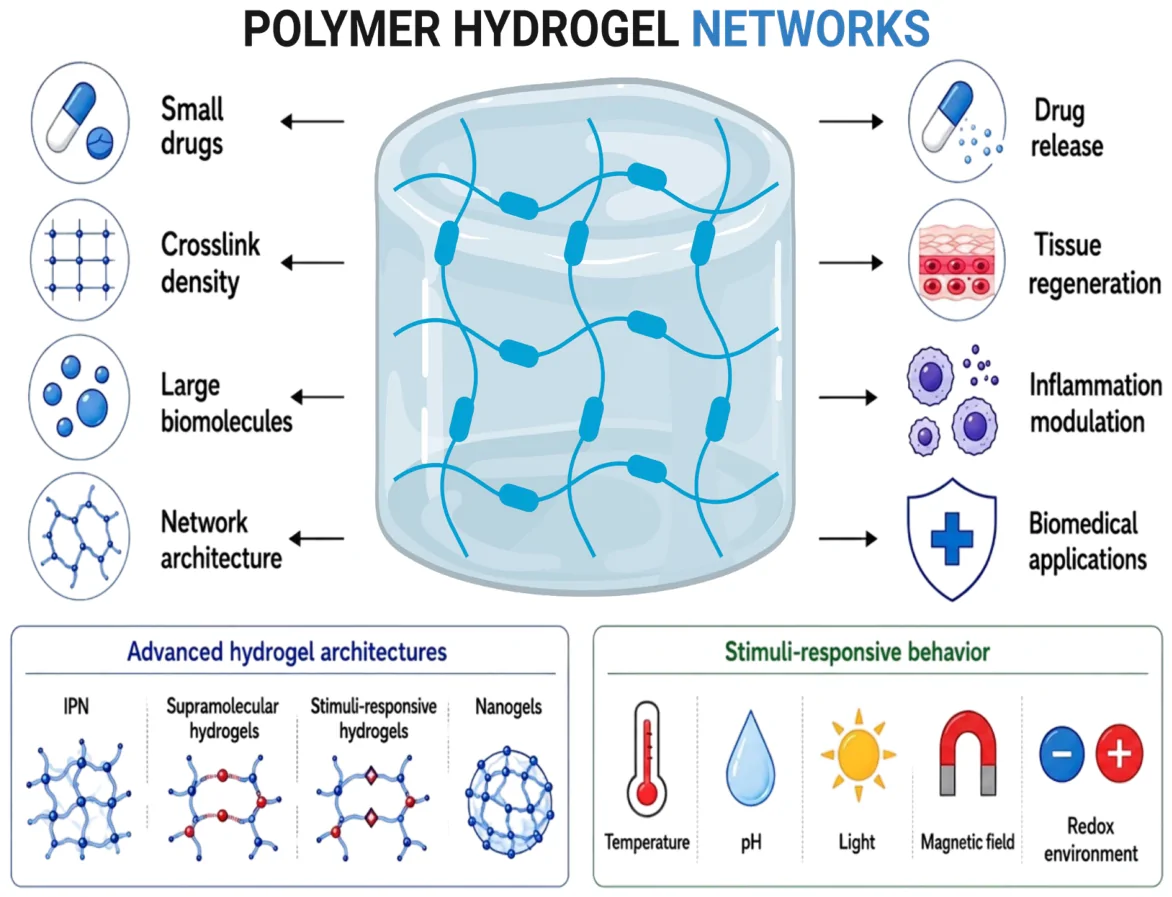
Schematic of polymer hydrogel networks as tunable diffusion–retention matrices, where mesh size, crosslinking, and swelling regulate the transport and controlled interactions of biomolecules, providing a physicochemical framework for emerging antiviral biointerface applications. Created in BioRender. Rizo, J. (2026) https://BioRender.com/2fde80k (accessed on 20 July 2026).

Polymeric hydrogels are highly hydrated three-dimensional networks that have evolved from passive scaffolds into programmable matrices for diffusion, retention, and controlled release [48,49,50]. Their relevance in complex biological environments, such as chronic wounds, lies in their ability to decouple mass transport from mechanical properties through rational network design, enabling simultaneous tissue compatibility and functional performance. This makes hydrogels particularly suitable as adaptive biointerfaces for regulating the transport of biomacromolecules while providing a promising platform for emerging antiviral applications [62,63,64]. The structural tunability of hydrogel networks and their role as diffusion–retention matrices are schematically illustrated in Figure 3.

### 3.2. Mesh Architecture, Porosity, and Diffusion Regimes

The transport behavior within hydrogels is primarily governed by mesh size distribution, pore architecture, and network connectivity. Recent studies indicate that classical Gaussian models underestimate structural heterogeneity, whereas Weibull-type distributions more accurately describe real hydrogel networks, with direct implications for macromolecular diffusion and NP transport [48,49,65].

Key parameters such as crosslink density, mesh size (ξ), polymer composition, swelling ratio, and the incorporation of functional additives collectively determine the balance between diffusion and retention. Increased crosslinking reduces diffusivity while enhancing retention of large species, whereas larger mesh sizes favor transport but decrease confinement [65,66,67,68,69,70,71,72,73]. Importantly, confinement effects within structured channels can induce subdiffusive behavior, particularly in systems with reduced pore size or elongated geometries, where hydrodynamic interactions and matrix–solute coupling become dominant [49,74,75]. Table 2 summarizes the key structural parameters governing diffusion and retention in hydrogel networks, highlighting their interdependent effects on mass transport behavior.

### 3.3. Interpenetrating and Hybrid Network Architectures

To further refine transport control, advanced architectures such as interpenetrating polymeric networks (IPN), semi-IPN, and supramolecular hydrogels have been developed. These systems enable independent tuning of mechanical properties, charge distribution, and diffusion pathways. For instance, PHEMA/PDMAM IPNs allow modulation of ξ and drug release profiles, achieving both rapid and sustained delivery depending on network configuration [76,77].

Thermosensitive semi-IPN systems, such as PNIPAM-based hydrogels, introduce dynamic regulation of swelling and retention as a function of temperature, polymer concentration, and molecular weight, enabling adaptive transport behavior [70,71]. Emerging upper critical solution temperature (UCST)-type hydrogels further broaden the spectrum of thermoresponsive biomaterials. Unlike lower critical solution temperature (LCST) systems, UCST hydrogels undergo phase transitions that can also regulate swelling, diffusion, retention, and controlled release, thereby providing complementary strategies for transport modulation. Although their biomedical application remains less mature than that of LCST-based systems, recent reviews highlight their promising potential for responsive drug delivery and adaptive biomaterial design [78,79,80]. Similarly, supramolecular hydrogels based on nucleoside assemblies exhibit tunable diffusion controlled by ionic strength and molecular packing, highlighting the role of reversible interactions in regulating mass transport [75,81].

### 3.4. Stimuli-Responsive and Nanoscale Hydrogel Systems

The integration of stimuli-responsive elements has transformed hydrogels into intelligent systems capable of spatiotemporal control over transport processes. Hydrogels responsive to pH, temperature, light, or redox conditions can dynamically modulate pore structure and diffusion pathways in response to environmental cues, a feature particularly relevant in heterogeneous wound microenvironments [65,69,82,83,84,85]. At the nanoscale, nanogels extend these capabilities by providing enhanced surface area and rapid responsiveness. Hybrid systems incorporating metallic NPs or photothermal agents enable externally triggered release, such as near-infrared (NIR)-induced heating, which accelerates molecular transport and enhances therapeutic efficacy [65,69,83,86,87].

### 3.5. Implications for Multifunctional Biomedical Platforms

Collectively, these advances establish hydrogels as highly versatile platforms for biomedical applications, including drug delivery, inflammation control, cancer therapy, and tissue regeneration. Their ability to regulate hydration, porosity, degradation, and responsiveness supports their use across multiple administration routes and clinical contexts [69,83,85,87,88,89]. Importantly, from a structure–function perspective, hydrogel networks can be engineered as selective diffusion–retention matrices, where controlled confinement, surface interactions, and adaptive transport behavior converge. This capability provides the foundational physicochemical framework for integrating MOFs into hybrid hydrogel systems, with the potential to enable the capture and stabilization of viral particles in complex biological environments through controlled transport, selective molecular interactions, and adaptive biomaterial design [62,64].

## 4. MOFs as Affinity Domains for Viral Envelope Interaction

MOFs have emerged as highly versatile porous materials for antiviral and antimicrobial applications owing to their tunable porosity, large surface area, and chemically active metal centers. Although most reported antiviral studies have focused on environmental decontamination, biosensing, or antiviral coatings rather than wound dressings, these findings provide a valuable physicochemical foundation for exploring future hydrogel–MOF biointerfaces for multifunctional wound care [69,83,85,87,88,89]. Although these antiviral applications remain largely outside the context of chronic wound management, they provide important physicochemical principles that may inspire the rational design of multifunctional hydrogel–MOF wound dressings with emerging antiviral biointerface functions. Their multifunctional character makes them particularly attractive for integration into advanced biomedical materials aimed at pathogen sequestration and infection control.

Viruses behave similarly to nanoscale protein colloids, exhibiting surface charge distributions and isoelectric points that strongly influence their interaction with solid interfaces. Since most enveloped viruses possess an overall negative surface charge under physiological conditions (pH 7.4), electrostatic attraction toward positively charged MOF surfaces constitutes one of the principal mechanisms of viral adsorption. However, virus–MOF interactions are not limited to electrostatics and may also involve van der Waals forces, π–π interactions, acid–base interactions, hydrophobic effects, and short-range surface forces [90,91].

Consequently, the adsorption efficiency depends not only on MOF surface chemistry but also on pore size distribution, surface charge density, and structural topology. Importantly, pore dimensions comparable to viral size favor confinement and immobilization within the porous network. This size-matching effect transforms MOFs into selective affinity domains capable of concentrating virions through steric confinement and interfacial interactions. In this context, MOFs function not only as adsorption materials but also as nanoscale trapping architectures that increase viral residence time and interaction probability [91].

Beyond structural properties, the chemical composition of MOFs plays a decisive role in antiviral activity. Metal ions exhibit strong affinity toward biomolecules containing electron-donating groups, allowing interactions with viral proteins, lipid envelopes, and nucleic acids. These interactions can alter viral structural stability, disrupt enzymatic processes, or induce oxidative stress [92]. Metal ions may coordinate with nitrogen-containing residues in proteins or phosphate groups in DNA/RNA, while environmental pH regulates their solubility, redox behavior, and biological accessibility [92].

The antiviral mechanisms of MOFs are therefore closely associated with their metallic components. Cu(II)-based systems can induce oxidative damage to viral genetic material and inhibit enzymatic activity, whereas Ag(I), Au(I), Pd(II), and Pt(II) species interact with sulfhydryl-containing proteins and disulfide-rich regions within viral envelopes [92]. Similarly, Co(III)-based complexes may interfere with zinc-finger proteins essential for viral replication through interactions with histidine residues and redox-mediated protein destabilization. These mechanisms collectively impair viral entry, replication, and structural integrity.

In enveloped viruses, Ag(I)-containing systems are particularly relevant because they can bind glycoprotein domains within the viral membrane, disrupt membrane-associated proteins, and interfere with viral internalization pathways. Meanwhile, photocatalytic MOFs capable of generating ROS represent an additional antiviral strategy. ROS such as superoxide radicals (O_2_^−^) and hydroxyl radicals (•OH) can oxidatively damage viral proteins, lipids, and nucleic acids, ultimately preventing replication and infectivity [92].

A comparative evaluation of representative MOF families highlights that their suitability for hydrogel-based biomedical applications depends on achieving an appropriate balance among structural stability, biological compatibility, functional activity, and translational feasibility. Zirconium-based MOFs, particularly UiO-type structures, are among the most promising candidates due to the high connectivity of Zr_6_ oxo clusters, excellent hydrolytic stability, low toxicity, favorable biocompatibility, and facile post-synthetic functionalization, making them especially attractive for incorporation into hydrated polymeric matrices and long-term regenerative applications [93,94,95,96]. Nevertheless, their relatively limited intrinsic antimicrobial activity often requires surface functionalization or combination with additional therapeutic components. Titanium-based MOFs also exhibit favorable biocompatibility and high chemical stability while providing photocatalytic activity capable of generating ROS under appropriate activation conditions. However, their antiviral performance generally depends on external light stimulation, and their biomedical application in hydrogel wound dressings remains comparatively underexplored because of the limited number of biological validation studies [96,97,98]. Copper- and silver-based MOFs exhibit strong intrinsic antimicrobial activity and potential antiviral properties through controlled metal-ion release, redox activity, and oxidative stress-mediated mechanisms. Nevertheless, excessive Cu^2+^ or Ag^+^ release may induce cytotoxicity and biosafety concerns, requiring careful optimization of ion concentration and degradation kinetics before clinical translation [90,95,99,100]. Zinc-based MOFs, particularly ZIF-8, provide a balanced combination of antibacterial activity, cytocompatibility, angiogenic potential, and regenerative performance through Zn^2+^-mediated biological functions, although their hydrolytic stability under physiological conditions is generally lower than that of zirconium-based frameworks, limiting their long-term applications [10,101,102]. Overall, no single MOF family can currently be considered universally optimal for hydrogel-based wound dressings. Instead, the selection of an appropriate MOF should be guided by the intended biomedical application and should balance framework stability, controlled ion release, biological activity, cytocompatibility, and long-term translational feasibility. Additional comparative studies remain necessary to establish the relative clinical value of different MOF families in multifunctional hydrogel wound dressings.

Among the most studied antiviral MOFs, zirconium-based structures such as UiO-66 and UiO-67 have demonstrated strong affinity toward SARS-CoV-2 proteins. Interestingly, structural geometry significantly influences adsorption behavior, as the octahedral morphology of UiO-67 appears more favorable for coronavirus protein confinement than the spherical structure of UiO-66, inducing conformational alterations in viral proteins [103]. Other MOFs with relevant structural architectures include HKUST-1, UiO-66-NH_2_, MIL-101(Cr), Fe(III)-MOF-5, and UL1MOF-6. In parallel, photocatalytic systems such as MIL-125(Ti)-NH_2_ and MIL-177(Fe) exhibit antiviral activity under UV irradiation through ROS generation, leading to oxidative disruption of viral capsids and inhibition of viral internalization [103].

**Figure 4:**
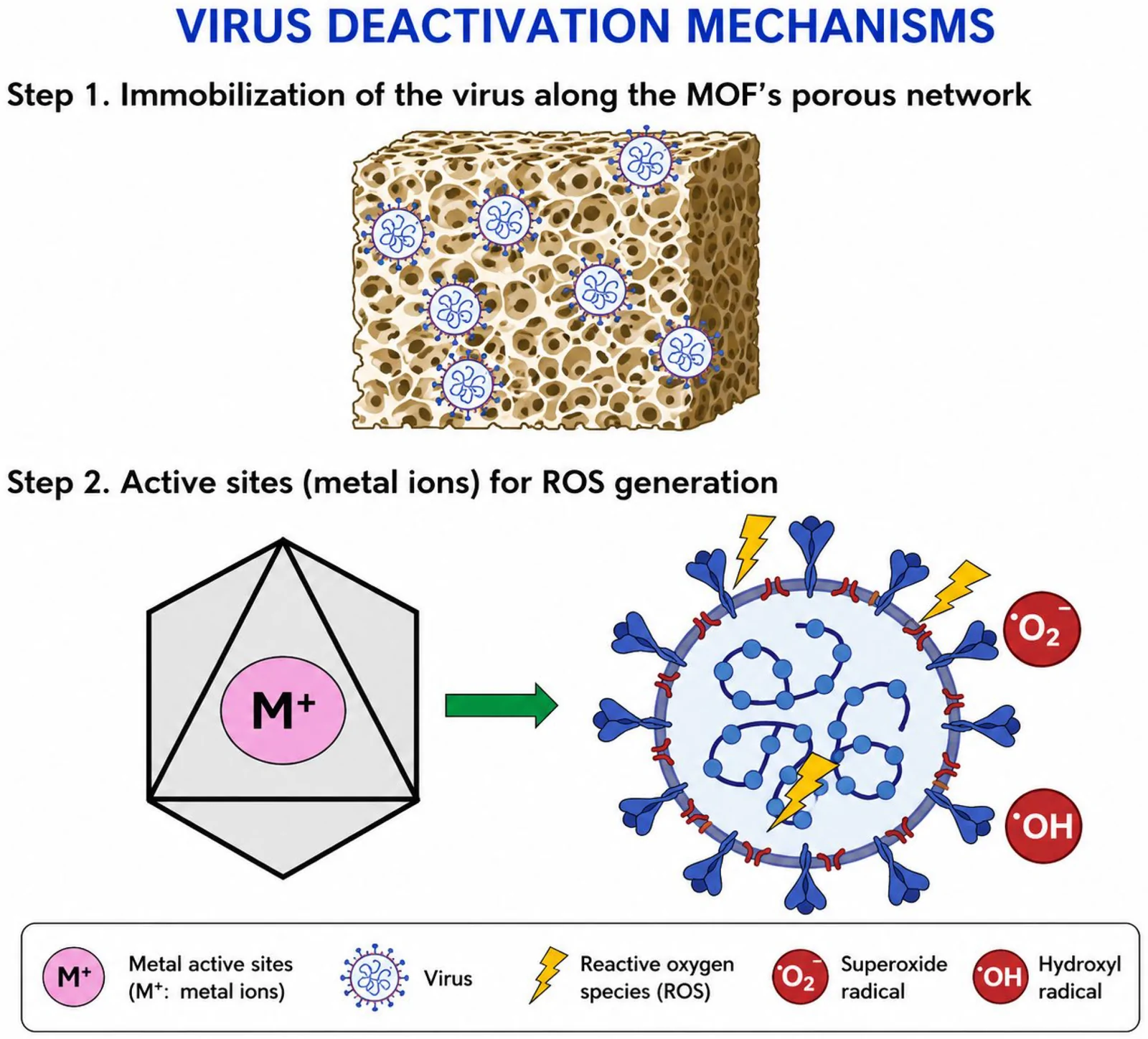
Mechanisms of viral adsorption and inactivation mediated by MOFs. Created in BioRender. Rizo, J. (2026) https://BioRender.com/ul6oqzm (accessed on 20 July 2026).

Despite these advances, relatively few studies have explored MOFs specifically as viral encapsulation or sequestration agents. In these systems, the porous framework acts as a precision confinement network capable of physically immobilizing virions, while the metallic active sites may simultaneously induce localized oxidative or chemical inactivation. This dual-action mechanism (Figure 4)—combining structural confinement with ROS-mediated antiviral activity—suggests that MOFs constitute promising affinity domains for investigating selective virus–material interactions. Consequently, the antiviral properties of MOFs provide a valuable physicochemical foundation for the future development of multifunctional hydrogel–MOF wound dressings incorporating emerging antiviral biointerface functions. Table 3 summarizes representative MOF-based antiviral platforms and illustrates how framework chemistry, surface affinity, and porous architecture contribute to virus–material interactions that may inspire future hydrogel–MOF biointerface design.

## 5. Structure–Property Relationships in Hydrogel–MOF Hybrid Polymer Composites

Hydrogel–MOF hybrid composites integrate hydrated polymeric networks with porous MOFs, generating multifunctional systems whose performance is governed by structure–property relationships across molecular, nano-, and microscale domains [108]. The rational organization of MOFs within polymer matrices enables precise modulation of mechanical behavior, mass transport, interfacial interactions, and biological functionality, thereby expanding the applicability of these materials in advanced biomedical applications [109].

MOFs contribute high surface area, tunable pore architectures, and chemically active metal sites capable of interacting with biomolecules and, potentially, viral particles. through electrostatic interactions, coordination bonding, hydrophobic interactions, and surface affinity effects. However, their intrinsic brittleness and limited processability restrict direct implementation under physiological conditions [110]. To overcome these limitations, hydrogel–MOF hybrid systems have emerged as adaptive composite platforms that combine the structural flexibility and hydration capacity of hydrogels with the selective adsorption and functional porosity of MOFs [32].

Within these composites, the hydrogel network acts as a mechanically compliant and biocompatible matrix that supports swelling, diffusion control, and tissue integration, while embedded MOFs introduce localized affinity domains for adsorption and controlled release. Consequently, the final performance of the material depends strongly on parameters such as MOF dispersion, pore hierarchy, interfacial compatibility, and hydrogel crosslinking density [111].

The interface between both components is particularly critical for determining functional stability. Strong interfacial interactions—including hydrogen bonding, electrostatic attraction, or covalent coupling—enhance mechanical integrity, prevent particle leaching, and improve stress transfer throughout the composite. In contrast, weaker interactions may favor accelerated release kinetics but compromise long-term structural stability [112]. Therefore, controlling interfacial chemistry becomes essential for balancing transport properties, adsorption performance, and regenerative functionality.

Importantly, hydrogel–MOF composites exhibit synergistic behavior that cannot be achieved by either component independently. The hydrogel phase regulates hydration, viscoelasticity, and diffusion pathways, whereas MOFs provide nanoscale confinement, selective affinity sites, and stimuli-responsive functionality. Through controlled adjustment of MOF loading, surface functionalization, and network architecture, these systems can be engineered to achieve tailored mass transport, enhanced mechanical properties, and selective biomolecular interactions [111,112].

**Figure 5:**
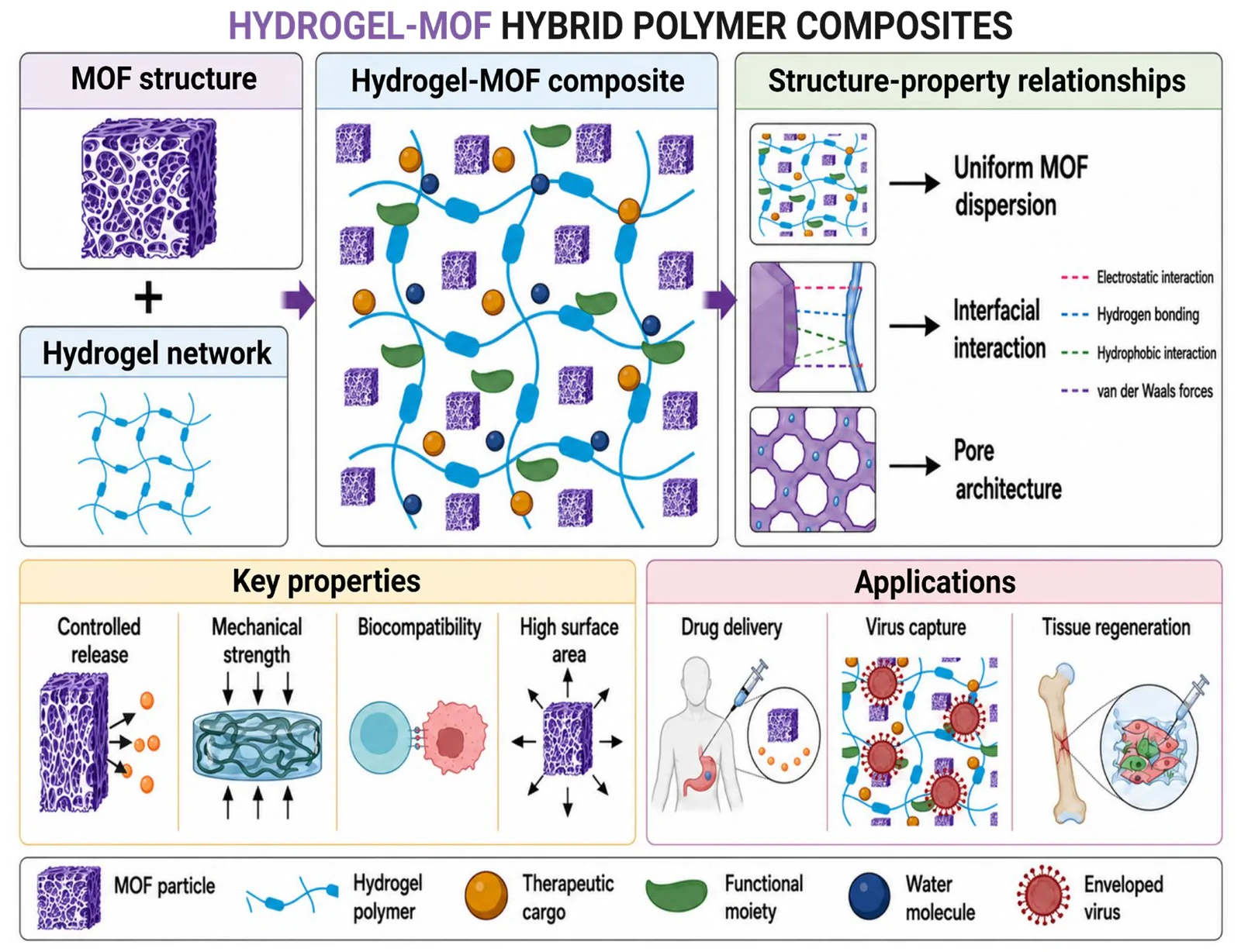
Design strategies and structure–function performance of hydrogel–MOF hybrid composites. Created in BioRender. Rizo, J. (2026) https://BioRender.com/3spdjhv (accessed on 20 July 2026).

From a structure–function perspective, the interplay between nanoscale MOF properties and macroscale hydrogel behavior represents a key design strategy for multifunctional materials capable of simultaneously supporting tissue regeneration while providing physicochemical platforms for potential pathogen sequestration. In particular, pore hierarchy, network connectivity, and surface chemistry collectively influence adsorption kinetics, molecular diffusion, potential viral adsorption and immobilization mechanisms [111,112]. These relationships are especially relevant for chronic wound environments, where dynamic transport regulation and selective bioadsorption are required under complex physicochemical conditions [83,111]. Figure 5 illustrates the structure–property relationships governing hydrogel–MOF hybrid composites. The integration of MOF particles within a hydrogel network generates multifunctional systems in which controlled release, molecular interactions, and mechanical performance emerge from the synergy between the hydrated polymer matrix and the porous MOF domains. Key parameters such as MOF dispersion, pore hierarchy, and interfacial interactions determine transport behavior and functional performance, drug delivery, viral adsorption and sequestration, and tissue regeneration.

Table 4 summarizes representative hydrogel–MOF hybrid systems, highlighting how structural engineering strategies—including dual-network architectures, in situ MOF growth, and surface-functionalized composites—directly influence mechanical stability, adsorption behavior, controlled release, and biomedical performance. Collectively, these studies demonstrate that rational structural design is fundamental for developing next-generation hydrogel–MOF platforms with integrated regenerative functions with emerging antiviral biointerface functions.

## 6. Mechanistic Pathways of Enveloped Virus Bioadsorption

The bioadsorption of enveloped viruses within hydrogel–MOF hybrid systems is proposed to be governed by interconnected physicochemical and mechanical mechanisms that may collectively regulate viral diffusion, retention, and stabilization within the material architecture [26,118]. Unlike conventional adsorption systems, hydrogel–MOF composites provide a dynamic and hierarchically structured environment where pore geometry, interfacial roughness, swelling behavior, and MOF distribution influence mass transport and virus–material interactions. Experimental studies have demonstrated that hydrogels regulate molecular diffusion while MOFs provide high-surface-area adsorption domains [27,119]. However, the integration of these mechanisms into multifunctional wound dressing systems specifically intended for enveloped virus sequestration remains largely conceptual and requires further experimental validation.

**Figure 6:**
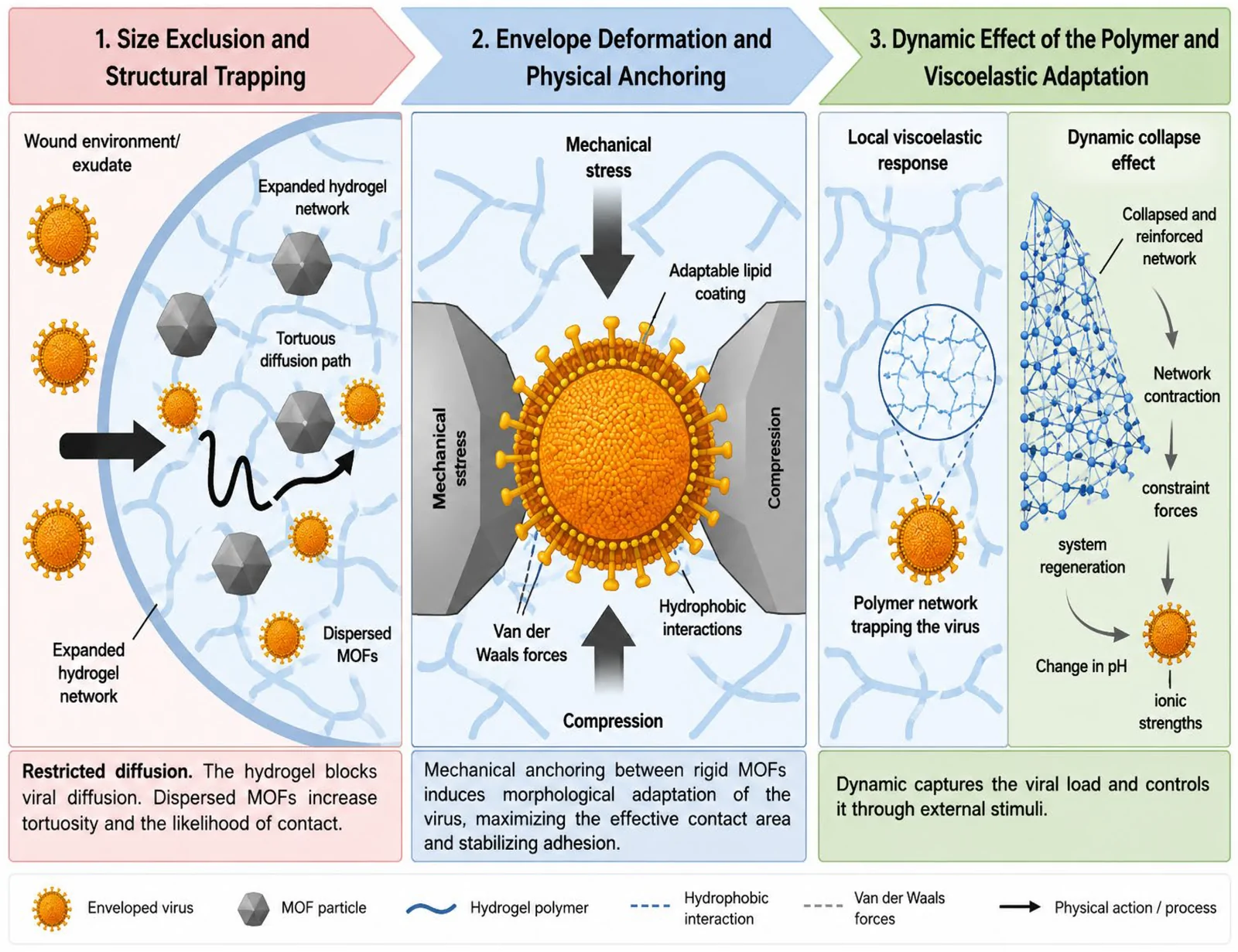
Mechanical pathways of viral bioadsorption in hydrogel–MOF hybrid composites. Created in BioRender. Rizo, J. (2026) https://BioRender.com/i8we5sv (accessed on 20 July 2026).

As illustrated in Figure 6, the mechanistic framework proposed in this review combines experimentally supported physicochemical interactions with engineering concepts that may guide the future design of antiviral hydrogel–MOF composites. Within this framework, viral capture refers to the initial adsorption of viral particles onto hydrogel–MOF interfaces, whereas viral immobilization describes the subsequent restriction of viral mobility resulting from structural confinement and interfacial interactions. Table 5 summarizes the principal structure–function relationships governing both regenerative performance and the proposed mechanisms of viral bioadsorption.

### 6.1. Size Exclusion and Structural Trapping

Enveloped viruses generally exhibit diameters ranging from approximately 80 to 200 nm, requiring them to traverse hydrated polymeric networks before reaching adsorption-active domains [120]. Experimental studies have shown that hydrogel architecture strongly influences molecular diffusion and transport properties, whereas incorporation of dispersed NPs or porous fillers increases transport tortuosity by modifying available diffusion pathways [17].

Based on these experimentally established transport phenomena, it is proposed that the incorporation of MOFs within hydrogel matrices may increase the residence time of viral particles by generating confined interstitial regions and reducing effective diffusivity [121]. Consequently, prolonged residence within the composite could increase the probability of virus–surface interactions with adsorption-active domains.

Accordingly, hierarchical pore organization generated by hydrogel–MOF interfaces may function as a selective physical barrier capable of slowing viral transport while favoring localized accumulation and retention. Although this mechanism is physically plausible and supported by transport theory, direct experimental validation in hydrogel–MOF wound dressing systems remains limited.

**Table 5:** Mechanistic bioadsorption pathways and structure–function design parameters in hydrogel–MOF hybrid systems for enveloped virus sequestration.

Mechanistic Pathway	Key Structural Parameter	Proposed Mechanism of Viral Retention	Regenerative Implication	Hydrogel–MOF Design Strategy
Matrix-controlled diffusion	Hydrogel pore size/MOF loading [17,121]	Is proposed to increase virus–surface collision frequency through restricted diffusion and tortuous transport pathways	Preserves hydrated microenvironment and controlled permeability [17,121]	Modulation of crosslinking density and homogeneous MOF dispersion [17,121]
Spatial confinement and retention	Mechanical stiffness/domain heterogeneity [17,121,122,123,124,125,126]	Is proposed to enhance physical confinement and prolong viral residence time within the composite	Maintains structural stability during tissue remodeling [17,121,122,123,124,125,126]	Control of viscoelastic modulus and hierarchical network architecture [17,121,122,123,124,125,126]
Envelope deformation and interfacial anchoring	Interfacial roughness/network flexibility [17,120,121,122,127]	Is proposed to promote conformational adaptation of the viral envelope and multisite surface interactions	Balances flexibility and mechanical integrity for tissue compatibility [17,120,121,122,127]	Optimization of hydrogel elasticity and MOF interfacial exposure [17,120,121,122,127]
Viscoelastic adaptation	Polymer composition and chain mobility [96,128,129]	Is proposed to reduce viral mobility through dynamic polymer rearrangement and frictional stabilization	Supports ECM-mimetic behavior and cellular migration [96,128,129]	Use of collagen, chitosan, or dynamic supramolecular networks [96,128,129]
Stimuli-responsive dynamic trapping	Swelling behavior/pH- or hydration-responsive contraction [96,128,129,130,131]	Is proposed to induce reversible expansion–collapse transitions that may contribute to prolonged viral confinement	Enables controlled release and adaptive wound response [96,128,129,130,131]	Development of stimuli-sensitive smart hydrogels integrated with functional MOFs [96,128,129,130,131]
Electrostatic viral sequestration	Surface charge density/functional groups [127,130,131,132,133]	Is proposed to promote Coulombic attraction between negatively charged viral envelopes and cationic domains	Excessive charge may compromise cytocompatibility [127,130,131,132,133]	Controlled incorporation of cationic polymers or metal-active MOFs [127,130,131,132,133]
Hydrophobic partitioning	Hydrophilic–hydrophobic balance [122,123,124]	Is proposed to facilitate interaction between lipid viral envelopes and hydrophobic MOF/polymer domains	Maintains moisture balance while enhancing adsorption [122,123,124]	Engineering amphiphilic polymer–MOF interfaces [122,123,124]
Ligand-mediated molecular recognition	Surface functionalization with peptides, antibodies, or aptamers [134,135,136]	Is proposed to enable selective binding toward viral envelope proteins	Improves specificity while minimizing nonspecific interactions [134,135,136]	Biofunctionalization of MOF surfaces and hydrogel matrices [134,135,136]

Note: The proposed mechanisms of viral retention presented in third column represent a conceptual framework inferred from the reported physicochemical properties and structure–function relationships of hydrogel–MOF composites. These mechanisms have not yet been experimentally demonstrated specifically for enveloped virus adsorption and should be interpreted as hypothesis-driven design principles to guide future investigation.

### 6.2. Envelope Deformation and Physical Anchoring

A distinctive feature of enveloped viruses is the presence of a deformable lipid membrane capable of adapting to irregular material surfaces [122,123]. Experimental observations from virus–surface interaction studies indicate that soft viral envelopes can undergo conformational adaptation upon contact with nanostructured materials, increasing the effective contact area and facilitating stabilization through weak intermolecular interactions, including hydrogen bonding, van der Waals forces, and hydrophobic interactions [124,125].

Based on these observations, it is proposed that heterogeneous interfaces formed between rigid MOF particles and compliant hydrogel domains may act as nanoscale anchoring sites. Partial conformational adaptation of viral envelopes to these interfaces could increase interfacial friction and reduce detachment probability under physiological flow conditions [126,127].

Consequently, envelope deformation and physical anchoring may contribute to prolonged viral immobilization within hydrogel–MOF composites. Nevertheless, these mechanisms have not yet been directly demonstrated in multifunctional wound dressing systems and should therefore be interpreted as mechanistic hypotheses derived from current knowledge of virus–material interactions.

### 6.3. Dynamic Retention Induced by Polymer Responsiveness

One of the defining characteristics of hydrogel-based systems is their dynamic responsiveness to environmental stimuli such as hydration, ionic strength, temperature, and pH [133]. Experimental studies have demonstrated that swelling and contraction modify hydrogel pore architecture and regulate molecular transport within polymeric networks [130,131,133].

Based on these well-established hydrogel properties, it is proposed that environmental fluctuations may influence viral confinement by facilitating penetration during swelling and promoting partial mechanical immobilization during subsequent matrix contraction. Polymer chain rearrangement surrounding viral particles may further stabilize retention, while embedded MOFs could reinforce the composite architecture and reduce structural relaxation.

Such behavior may be particularly relevant under chronic wound conditions, where continuous variations in hydration, pH, ionic composition, and exudate composition occur [128]. Although these adaptive responses are well documented for hydrogel materials, their contribution to selective viral sequestration within hydrogel–MOF wound dressings remains hypothetical and awaits direct experimental confirmation [96,129].

Beyond mechanical confinement, hydrogel–MOF composites may also rely on multiple physicochemical interactions that synergistically contribute to viral adsorption [134]. Electrostatic attraction is considered one of the most plausible mechanisms because many enveloped viruses exhibit an overall negative surface charge under physiological conditions. Accordingly, positively charged functional groups present within hydrogels or MOF surfaces may promote virus–material interactions through Coulombic attraction [135].

Hydrophobic interactions may further enhance adsorption owing to the lipidic nature of viral envelopes. Likewise, incorporation of ligands such as peptides, antibodies, aptamers, or receptor-mimicking molecules could provide selective molecular recognition of viral envelope proteins [134,136]. These interaction mechanisms are supported by broader studies of virus–material interfaces; however, their combined implementation within multifunctional hydrogel–MOF wound dressings remains an emerging engineering concept rather than an experimentally established antiviral strategy.

Importantly, the antiviral mechanisms discussed throughout this section should be interpreted as future engineering opportunities inspired by the physicochemical properties of hydrogel–MOF systems rather than as clinically established functions of current wound dressings. Continued experimental validation will be essential to determine their translational relevance.

## 7. Dual Functional Performance: Tissue Regeneration Versus Viral Sequestration

Chronic and infected skin wounds remain a major clinical challenge due to the coexistence of impaired tissue repair, persistent inflammation, and microbial contamination within a highly dysregulated microenvironment [137]. Although bacterial infection remains the principal clinical concern, recent advances in antiviral biomaterials have prompted interest in exploring whether future wound dressings could also incorporate antiviral biointerface functions [138]. Consequently, next-generation biomaterials may eventually combine regenerative performance with complementary pathogen sequestration strategies.

Hydrogels have been extensively investigated for wound management because of their ability to maintain a moist microenvironment that promotes cell migration, reduces dehydration, and facilitates ECM remodeling [139,140,141]. Their high biocompatibility and structural resemblance to native ECM further support fibroblast proliferation, angiogenesis, and tissue regeneration [142]. Moreover, hydrogel physicochemical properties can be rationally engineered to incorporate antimicrobial activity, controlled release profiles, and pro-regenerative signaling mechanisms [143,144]. Despite these advances, most conventional hydrogel systems have primarily focused on bacterial control, whereas viral persistence and viral adsorption at wound-associated surfaces remain comparatively underexplored. Enveloped viruses, including coronaviruses and influenza viruses, possess lipid bilayer membranes decorated with glycoproteins that mediate host recognition and cellular internalization [145]. Beyond protecting the viral core, this lipid envelope governs interactions with biological and synthetic surfaces through electrostatic and hydrophobic mechanisms [146]. Importantly, enveloped viruses may remain viable on abiotic surfaces for prolonged periods, increasing the risk of indirect transmission in clinical environments [147,148]. These characteristics have motivated interest in exploring wound dressings capable not only of preventing bacterial colonization, but also of selectively interacting with and immobilizing viral particles.

Within this context, MOFs have emerged as promising components for exploring emerging antiviral biointerface applications due to their tunable porosity, high surface area, and adaptable surface chemistry [90,149,150]. Their porous architecture may facilitate virus–material interactions, including viral adsorption and potential sequestration through diffusion confinement and interfacial adsorption, functionalized surfaces enable while functionalized surfaces may enable selective interactions with viral proteins and lipid membranes. In addition, MOFs can incorporate bioactive metal ions capable of controlled release or ROS generation, contributing to viral inactivation and antimicrobial activity [110]. Their potential to act simultaneously as adsorption domains and therapeutic reservoirs further expands their biomedical relevance.

Nevertheless, integrating antiviral functionality into regenerative biomaterials introduces a critical design trade-off. Tissue regeneration requires highly biocompatible environments with minimal oxidative stress and controlled inflammatory responses [9]. Conversely, antiviral mechanisms often depend on ROS production, ion release, or strong surface interactions that may compromise fibroblast viability, keratinocyte proliferation, and ECM restoration [151]. For example, excessive concentrations of metal-based nanomaterials may induce oxidative damage and cytotoxicity despite their potent antimicrobial activity [152]. Therefore, optimizing the balance between antiviral efficacy and regenerative compatibility represents one of the principal challenges in hydrogel–MOF engineering.

**Figure 7:**
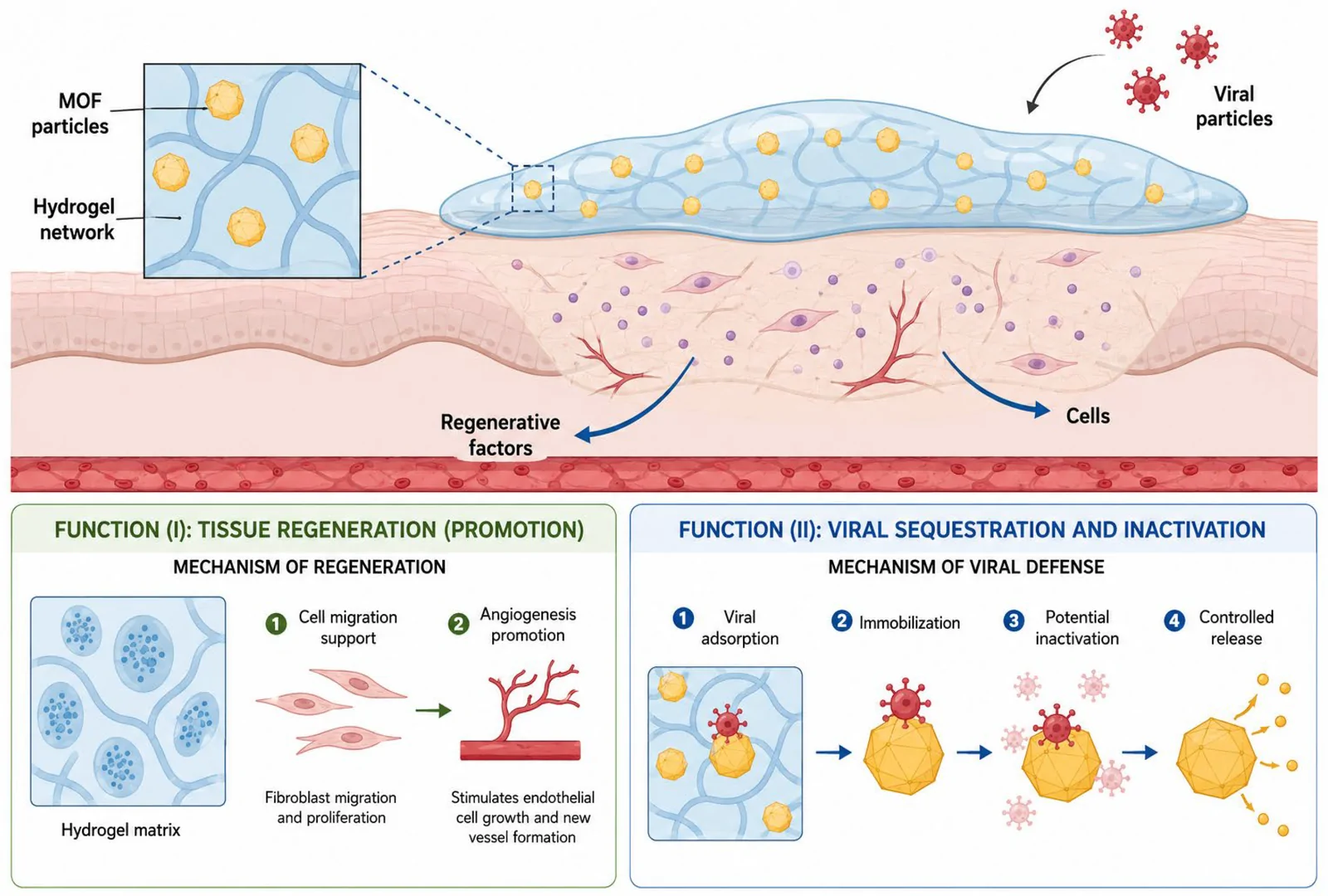
Dual-functional hydrogel–MOF composite for simultaneous tissue regeneration and enveloped virus adsorption and sequestration at the wound interface. Created in BioRender. Rizo, J. (2026) https://BioRender.com/qx4m79g (accessed on 20 July 2026).

Hybrid hydrogel–MOF composites offer a versatile strategy to reconcile these opposing requirements within a single multifunctional platform, as illustrated in Figure 7. In these systems, the hydrogel matrix provides a hydrated, cytocompatible, and mechanically compliant microenvironment favorable for tissue repair [153,154], while embedded MOFs may act as active domains for viral adsorption, controlled therapeutic release, and antimicrobial modulation [154], allowing simultaneous optimization. Importantly, the tunability of both components enables precise control over pore architecture, surface charge, swelling behavior, mechanical properties, and interfacial interactions, potentially allowing simultaneous optimization of regenerative and antiviral performance.

Overall, achieving an appropriate balance between regenerative performance and emerging antiviral functionality requires simultaneous optimization of several interdependent material parameters, including polymer composition, MOF loading, pore architecture, surface chemistry, swelling behavior, mechanical properties, and controlled ion release. These parameters collectively regulate cytocompatibility, viral adsorption efficiency, therapeutic release, oxidative stress, and structural stability, illustrating the fundamental structure–function trade-offs that must be considered during the rational design of multifunctional hydrogel–MOF wound dressings [9,149,151,152,153,154].

Taken together, hydrogel–MOF hybrid systems represent an emerging generation of multifunctional wound dressings capable of integrating tissue regeneration with selective viral adsorption and sequestration. Although fully optimized antiviral–regenerative platforms remain at an early stage of development, recent studies indicate that rational structure–function engineering can generate composites with complementary adsorption, antimicrobial, and regenerative properties. Importantly, the antiviral functions discussed throughout this section should be regarded as emerging engineering opportunities that require further experimental validation before clinical translation. Representative hydrogel–MOF systems exhibiting dual functional performance are summarized in Table 6. Importantly, the antiviral concepts discussed throughout this review should be interpreted as future engineering opportunities inspired by the physicochemical properties of hydrogel–MOF systems rather than as clinically established functions of current wound dressings. Continued experimental validation will be essential to determine their translational relevance.

## 8. Design Parameters for Selective Viral Bioadsorption in Polymeric Dressings

The rational engineering of polymeric wound dressings for potential viral bioadsorption requires precise control over the physicochemical interactions established between the biomaterial, the wound microenvironment, and viral particles. Unlike conventional dressings primarily focused on moisture retention and bacterial control, next-generation hydrogel-based systems should integrate emerging antiviral biointerface functions, tissue compatibility, and regenerative functionality within a single therapeutic platform [12,159]. Since the infectivity and persistence of enveloped viruses are strongly influenced by their surface chemistry, structural stability, and environmental adaptability, the design of antiviral dressings must be tailored according to both the biological characteristics of the pathogen and the dynamic conditions of chronic wounds.

Among the most critical parameters, moisture regulation remains fundamental for maintaining cellular viability, facilitating fibroblast migration, and supporting ECM remodeling during healing [160]. Hydrogels derived from natural polymers, including chitosan, alginate, collagen, and hyaluronic acid, are particularly attractive due to their high water uptake capacity, biocompatibility, and structural similarity to native tissues [39,161,162]. Beyond exudate management, these hydrated matrices also provide a diffusion-permissive environment that may facilitate viral transport toward antiviral affinity domains incorporated into the dressing, thereby enhancing opportunities for virus–material interactions.

Surface charge represents another key determinant governing viral adsorption efficiency. Most enveloped viruses exhibit negatively charged surfaces under physiological conditions; therefore, the incorporation of cationic groups such as ammonium, guanidinium, or positively charged MOFs containing Zn^2+^, Ag^+^, or Cu^2+^ may significantly enhance electrostatic attraction and promote potential viral sequestration [162,163,164,165]. However, excessive cationic density may disrupt local pH, induce oxidative stress, or compromise fibroblast viability, highlighting the importance of balancing antiviral activity with cytocompatibility [166,167]. In this context, hydrogel–MOF systems offer a tunable platform where surface charge density and ion release kinetics can be rationally tuned.

Pore architecture and network organization also may play important roles in selective viral capture. ξ, crosslinking density, and hierarchical porosity directly influence fluid transport, diffusion kinetics, and virion transport and confinement within the polymeric matrix. While smaller pores may improve retention efficiency and increase virus–material interactions, excessively dense networks may impair oxygen permeability and cellular infiltration, ultimately compromising tissue regeneration [160,168]. Consequently, optimized porous architectures are required to simultaneously promote wound healing and selective pathogen sequestration.

Equally important is the balance between hydrophilic and hydrophobic domains within the dressing. Hydrophilic regions favor exudate absorption and maintain a regenerative microenvironment, whereas hydrophobic segments may contribute to destabilization of viral lipid envelopes through membrane interactions [169]. Nevertheless, excessive hydrophobicity may reduce water retention and impair tissue compatibility [170]. Therefore, finely tuned amphiphilic systems represent a promising strategy for achieving simultaneous antiviral and regenerative performance.

Mechanical stability further determines the clinical functionality of polymeric dressings. Materials intended for chronic wound management must exhibit adequate elasticity, flexibility, and structural resilience to withstand prolonged exposure to exudate and mechanical stress [171]. Hybrid hydrogel–MOF composites reinforced with nanostructured domains have demonstrated improved mechanical integrity, resistance to hydrolytic degradation, and prolonged functional performance while preserving hydrogel softness and conformability [16,172].

Finally, ligand-mediated functionalization constitutes one of the most advanced strategies for selective viral bioadsorption. The incorporation of antibodies, aptamers, sulfated polysaccharides, or receptor-mimicking ligands may enable highly selective interactions with viral glycoproteins, potentially facilitating targeted sequestration and inhibition of viral attachment to host cells [173,174,175,176,177]. Although these biomimetic approaches substantially enhance specificity, challenges associated with synthesis complexity, stability, and production cost remain important considerations for clinical translation.

**Figure 8:**
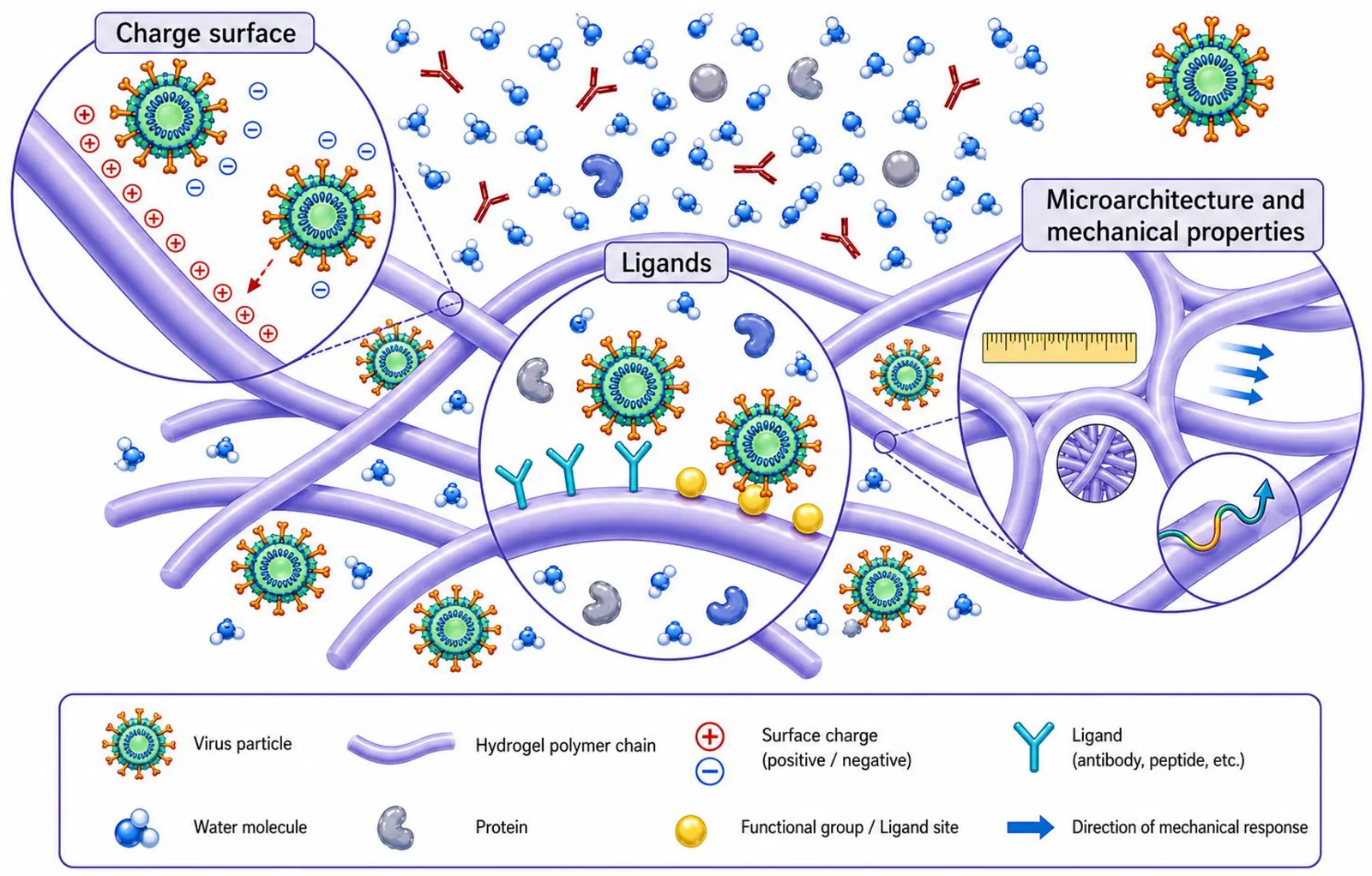
Schematic representation of the key design parameters governing selective viral bioadsorption in functionalized polymeric wound dressings, including electrostatic interactions, ligand-mediated viral recognition, pore and nanofiber architecture, hydrophilic–hydrophobic balance, and moisture-regulated antiviral microenvironments that collectively modulate viral sequestration and tissue regeneration (Created in https://www.canva.com) (accessed on 20 July 2026).

In summary, the design of multifunctional polymeric dressings for selective viral bioadsorption requires the synergistic integration of moisture regulation, electrostatic tuning, pore engineering, amphiphilic balance, mechanical reinforcement, and molecular recognition (Figure 8). Rather than acting independently, these design parameters are highly interdependent. For example, pore architecture and swelling behavior govern mass transport and accessibility to adsorption sites, surface charge and ligand-mediated functionalization may determine the specificity and strength of virus–material interactions, whereas mechanical stability and moisture regulation preserve structural integrity, cytocompatibility, and regenerative performance. Consequently, successful hydrogel–MOF systems require balanced optimization of all these parameters rather than maximizing any single property in isolation. Table 7 summarizes the principal design parameters governing selective viral bioadsorption in multifunction [16,159,161,162,163,164,165,166,167,168,169,170,171,172,173,174,175,176].

## 9. Current Limitations, Translational Challenges, and Future Perspectives

Despite the remarkable progress achieved in hydrogel–MOF hybrid systems for tissue regeneration and viral sequestration, several scientific and translational limitations continue to hinder their clinical implementation. One of the principal challenges lies in balancing antiviral performance with cytocompatibility. Strategies based on ROS generation, metal-ion release, or strong electrostatic interactions may effectively inactivate viral particles; however, excessive oxidative stress or uncontrolled ion concentrations can damage fibroblasts, keratinocytes, and surrounding healthy tissues, ultimately compromising wound healing outcomes [182,183]. Therefore, the precise regulation of antiviral activity while preserving regenerative functionality remains a critical design requirement.

Another important limitation concerns the structural stability and reproducibility of hydrogel–MOF systems under physiological conditions. Although MOFs provide high surface area and tunable adsorption properties, many frameworks exhibit hydrolytic instability, particle aggregation, or uncontrolled degradation in highly hydrated and protein-rich wound environments [184]. Similarly, the incorporation of MOFs may alter hydrogel swelling behavior, viscoelasticity, and mechanical integrity, affecting long-term performance and dressing durability [185]. Achieving homogeneous MOF dispersion and stable interfacial interactions within soft polymeric matrices therefore remains a major materials engineering challenge.

In this context, polymer selection becomes particularly important for optimizing the dual regenerative–antiviral performance of these systems. Natural polymers such as collagen, gelatin, alginate, hyaluronic acid, chitosan, and silk fibroin provide excellent biocompatibility, bioactivity, and ECM-mimetic properties that favor cell adhesion and tissue regeneration [15]. Chitosan and collagen are especially attractive due to their intrinsic positive surface charge and potential antiviral interactions [15]. However, natural polymers frequently exhibit poor mechanical resistance, rapid degradation, and batch variability [15,186,187]. Conversely, synthetic polymers including poly(vinyl alcohol) (PVA), PEG, poly(N-isopropylacrylamide) (PNIPAM), polyurethane, and polycaprolactone (PCL) offer improved mechanical stability, tunable degradation kinetics, and stimuli-responsive behavior [81,188]. The integration of natural and synthetic polymers into hybrid or interpenetrating networks therefore represents a promising strategy for balancing bioactivity, mechanical integrity, and controlled viral sequestration [189,190].

Selective viral detection within hydrogel–MOF environments also remains insufficiently explored. The highly hydrated, porous, and chemically active nature of these composites can interfere with conventional molecular biology techniques used for viral identification and quantification. Components such as polysaccharides, metal ions, proteins, or degradation byproducts may inhibit reverse transcription polymerase chain reaction (RT-PCR), affect nucleic acid extraction efficiency, or interfere with fluorescence-based biosensing platforms [32,134,191]. In addition, viral adsorption within hydrogel–MOF matrices may complicate viral recovery and accurate quantification. Consequently, future studies should develop standardized protocols for viral extraction, biosensing integration, and molecular analysis in complex biomaterial environments.

Despite the promising multifunctional characteristics of hydrogel–MOF composites, quantitative evaluations reported in the literature remain heterogeneous and are primarily focused on antibacterial activity, cytocompatibility, and wound repair rather than direct antiviral performance. For example, ZIF-8-containing hydrogel dressings have demonstrated antibacterial efficiencies above 98–99% against both *Escherichia coli* and *Staphylococcus aureus*, while maintaining high cytocompatibility in vitro [192]. Similarly, alginate–gelatin hydrogels incorporating ZIF-8 nanoparticles achieved approximately 99% bacterial inhibition, cell viability above 90%, and 89.4 ± 3.2% wound closure in vivo [193]. Cu/ZIF-8-integrated hydrogel platforms have also reported antibacterial efficiencies close to 99%, cell survival above 80%, enhanced angiogenic responses, and increased collagen deposition during wound repair [194]. Across representative experimental studies, wound closure rates generally range from approximately 92% to 97% within 7–14 days, substantially exceeding those observed in untreated controls [195,196,197,198]. Antibacterial activity has reached reductions of up to 4.1 log against Staphylococcus aureus and 3.4 log against Escherichia coli, while other systems have a chieved bacterial inhibition rates exceeding 90% [197,199,200]. Furthermore, Cu-MOF-based hydrogels have demonstrated approximately 1.5-fold greater collagen deposition and threefold higher angiogenesis than untreated controls, further supporting their regenerative potential [201]. Although ROS-scavenging activity and cytocompatibility have been consistently reported, quantitative values are not always presented using standardized methodologies across studies [195,202]. Importantly, quantitative antiviral performance metrics—including viral adsorption capacity, viral removal efficiency, and viral log-reduction values—remain scarcely reported for hydrogel–MOF wound dressings [90,105]. Therefore, establishing standardized antiviral evaluation protocols comparable to those currently used for antibacterial and regenerative assessments represents a critical requirement for future translational development.

**Table 8:** Representative quantitative performance metrics reported for hydrogel–MOF wound dressings in experimental studies.

Hydrogel–MOF System	Representative Performance Metrics	Quantitative Antiviral Metrics
GelMC/PVA-UiO-66-NH_2_@Que	92.02 ± 2.72% wound closure (14 days); enhanced angiogenesis [195]	Not reported
MOF/GOx-PP h ydrogel	93% wound closure (7 days); antibacterial activity against *E. coli* and *S. aureus* [196]	Not reported
OSD/CMC/Fe/PA3 + NIR	97.02% wound closure; 93.1% bacterial reduction in vivo [197]	Not reported
Cur@Cu-MOF hydrogel	~10^3^-fold reduction in bacterial burden; ~1.5-fold greater collagen deposition; ~3-fold higher angiogenesis [203]	Not reported
NHZ (ZIF-8) hydrogel	3.4-log reduction (*E. coli*) and 4.1-log reduction (*S. aureus*) [204]	Not reported
CMCS-CEBT hydrogel	94.5 ± 1.9% (*E. coli*) and 92.1 ± 1.7% *(S. aureus*) inhibition [200]	Not reported

Note. To date, no experimental hydrogel–MOF wound dressing has reported quantitative antiviral performance metrics (e.g., viral removal efficiency, viral adsorption capacity, or viral log-reduction values). This represents an important knowledge gap and highlights a key priority for future translational research.

From a translational perspective, scalable manufacturing and regulatory standardization remain major bottlenecks. Multifunctional hydrogel–MOF systems often require multistep synthesis, precise NP dispersion, sterilization compatibility, and reproducible physicochemical properties, increasing production complexity and cost [205,206]. Therefore, future development should emphasize Good Manufacturing Practice (GMP)-compatible fabrication routes, reproducible quality control strategies, and compliance with international standards for biomaterials and medical devices, including ISO 10993 guidelines for biocompatibility evaluation and standardized antiviral testing protocols [206,207,208]. Regulatory harmonization will be essential for accelerating clinical translation and ensuring safety, reproducibility, and industrial scalability. The principal priorities that should be addressed to facilitate the clinical translation of hydrogel–MOF wound dressings are summarized in Table 8.

To facilitate the clinical translation of hydrogel–MOF systems, a prioritized translational roadmap can be envisioned. The first priority should be the establishment of standardized and reproducible fabrication strategies, including precise control of MOF synthesis, particle distribution, polymer–MOF interactions, and batch-to-batch consistency. The second stage should involve systematic biological validation through advanced in vitro models and relevant in vivo studies to demonstrate biosafety, regenerative efficacy, antiviral performance, and long-term tissue compatibility. Once biological effectiveness is established, efforts should focus on scalable and GMP-compatible manufacturing processes, sterilization procedures, and robust quality-control protocols. Finally, regulatory alignment, standardized antiviral evaluation methods, and well-designed preclinical and clinical studies will be essential for successful translation into approved biomedical products.

This sequential framework emphasizes that clinical implementation requires not only improved material performance but also coordinated advances in manufacturing, regulation, and biological validation.

Future research should focus on the development of bioadaptive and stimuli-responsive hydrogel–MOF systems designed to dynamically modulate viral adsorption and capture, therapeutic release, and regenerative signaling according to wound microenvironment conditions. Advanced chemical engineering approaches based on supramolecular chemistry, dynamic covalent interactions, hierarchical pore design, and ligand-mediated targeting may significantly improve selectivity and multifunctionality [209,210]. Moreover, integrating computational modeling, artificial intelligence-assisted materials design, and high-throughput screening strategies could accelerate the optimization of next-generation antiviral wound dressings [211,212].

Hydrogel–MOF hybrid composites represent a highly promising platform for the development of multifunctional biomaterials that may simultaneously support tissue regeneration while incorporating emerging antiviral biointerface functions through selective viral adsorption and potential sequestration. Although important scientific, technological, and regulatory challenges remain, continued advances in polymer chemistry, nanotechnology, molecular biosensing, and biofabrication are expected to drive the emergence of clinically translatable systems for infection control and regenerative medicine.

## 10. Conclusions

Chronic wounds constitute highly dynamic physicochemical microenvironments where persistent inflammation, biofilm formation, ECM degradation, exudate accumulation, and redox imbalance collectively create conditions that may influence molecular transport, interfacial interactions, and biomaterial performance. In this review, chronic wounds are discussed as pathological microenvironments that provide a conceptual framework for exploring putative viral retention phenomena from a biomaterials engineering perspective, in which enveloped viruses may potentially interact with complex polymeric, biological, and microbial structures through electrostatic, hydrophobic, and structural confinement mechanisms. Although direct experimental evidence remains limited, this perspective expands the conventional understanding of chronic wound pathology beyond bacterial colonization and highlights opportunities for the future development of multifunctional biomaterials integrating established regenerative functions with hypothesized antiviral biointerface concepts.

Hydrogel–MOF hybrid systems emerge as particularly promising platforms because they integrate the hydrated and biocompatible nature of polymeric hydrogels with the high surface area, tunable porosity, and chemically active domains of MOFs. The synergistic interplay between both components may enable the rational modulation of diffusion, viscoelasticity, pore architecture, interfacial interactions, and molecular affinity, thereby potentially influencing viral adsorption, retention stability, controlled therapeutic release, and regenerative performance. Importantly, this review proposes that potential viral sequestration may involve not only electrostatic attraction but also size exclusion, envelope deformation, dynamic swelling behavior, and stimuli-responsive confinement within the hybrid network. These mechanisms should be regarded as hypothesis-driven structure–function relationships that warrant systematic experimental validation rather than as established antiviral mechanisms.

The present review further emphasizes that the future development of antiviral biointerface engineering will depend on advanced structure–function design strategies involving natural and synthetic polymers, hierarchical MOF architectures, ligand-mediated recognition, and bioadaptive responses to complex wound microenvironments. Nevertheless, major challenges remain regarding biosafety, manufacturing reproducibility, molecular detection of viruses within hydrogel–MOF matrices, large-scale production, and regulatory standardization. Future studies should systematically validate the proposed adsorption and sequestration mechanisms using clinically relevant enveloped viruses and standardized in vitro and in vivo models to establish causal structure–function relationships. Collectively, hydrogel–MOF composites represent a promising multifunctional biomaterial platform that may bridge regenerative medicine, antiviral materials science, supramolecular chemistry, and bioadsorption engineering. Their continued development may enable next-generation intelligent wound dressings that combine established regenerative performance with emerging antiviral biointerface functions while providing a rational, hypothesis-driven framework for future experimental and clinical investigation.

## Figures and Tables

**Table 1:** Chronic wound microenvironment factors and corresponding hydrogel–MOF design strategies for multifunctional wound healing.

Microenvironment Factor	Role in Pathology	Microenvironmental Implication	Hydrogel–MOF Design Strategy	Functional Outcome
Alkaline pH	Proteolysis and pathogen overgrowth [34,55,56,57,58]	Alters local physicochemical interactions and favors pathogen persistence [34,55,56,57,58]	pH-responsive hydrogel–MOF systems [34,55,56,57,58]	pH modulation and controlled therapeutic release [34,55,56,57,58]
Exudate	Persistent inflammation and protein-rich matrix [37,38,55,56]	Creates a protein-rich environment with restricted diffusion and sustained inflammation [37,38,55,56]	High-swelling, viscoelastic hydrogels [37,38,55,56]	Moisture balance, exudate management, and enhanced tissue regeneration [37,38,55,56]
Biofilm (EPS)	Antimicrobial resistance and persistence [14,56,57,59,60]	Persistent biofilm limits therapeutic penetration and maintains chronic infection [14,56,57,59,60]	MOFs with cationic and nucleic acid affinity [14,56,57,59,60]	Biofilm disruption and improved antimicrobial performance [14,56,57,59,60]
Hypoxia/ROS	Impaired healing and oxidative stress [4,31,54,55,56]	Sustains oxidative stress and delays tissue repair [4,31,54,55,56]	O_2_-releasing and nanozyme-like MOFs [4,31,54,55,56]	Microenvironment restoration, oxidative stress regulation, and tissue protection [4,31,54,55,56]
ECM/NETs	Structural degradation and chronic inflammation [51,52,56,61]	Promotes ECM remodeling and persistent inflammation [51,52,56,61]	ECM-mimetic and DNA-interactive MOFs [51,52,56,61]	Immunomodulation and improved wound repair [51,52,56,61]

Abbreviations: ECM, extracellular matrix; EPS, extracellular polymeric substances; MOF, metal–organic framework; NETs, neutrophil extracellular traps; O_2_, oxygen; ROS, reactive oxygen species.

**Table 2:** Key structural parameters governing diffusion and retention in polymer hydrogel networks.

Parameter	Description	Effect on Diffusion	Effect on Retention
Crosslink density	Degree of network connectivity [50,51,52]	↑ density → ↓ diffusion [50,51,52]	↑ retention of large molecules [50,51,52]
ξ	Effective pore size of the network [50,51,52,53]	↑ ξ → ↑ diffusion [50,51,52,53]	↓ ξ → ↑ retention [50,51,52,53]
Polymer composition	Chemical functionality and polarity [50,51,53,54]	Modulates matrix–solute interactions [50,51,53,54]	Enables selective retention [50,51,53,54]
Swelling ratio	Water uptake capacity [50,51,52,55,56,57]	↑ swelling → ↑ diffusion [50,51,52,55,56,57]	↓ swelling → ↑ retention [50,51,52,55,56,57]
Functional additives (NPs, MOFs, bioactives)	Incorporated nanophases or active agents [54,55,58,59,60,61]	Can enhance or restrict transport pathways [54,55,58,59,60,61]	Promotes targeted or affinity-driven retention [54,55,58,59,60,61]

Abbreviations: ξ, hydrogel mesh size; NPs, nanoparticles; MOFs, metal–organic frame-works. Symbols: ↑ indicates an increase or promotion; ↓ indicates a decrease or reduction; → indicates results in or leads to.

**Table 3:** Representative MOF-based systems explored for viral sequestration, adsorption, and antiviral surface interactions through porous confinement and physicochemical affinity mechanisms.

MOF System	Structural/Chemical Features	Target Virus	Mechanism of Viral Interaction	Functional Outcome
MIL-53(Al)/cellulose acetate composite	Al-based porous framework integrated into cellulose acetate beads [27]	HIV [27]	Hydrogen bonding and surface adsorption within porous domains [27]	High viral capture efficiency (99.93%) and virion immobilization [27]
UiO-66	Zr-based MOF functionalized with folic acid, tenofovir, and nystatin [104]	SARS-CoV-2 [104]	Interaction with glycosylated and non-glycosylated viral proteins [104]	Enhanced viral adsorption and multifunctional antiviral activity [104]
UiO-66-NH_2_	Amino-functionalized Zr-based MOF [104]	SARS-CoV-2 [104]	Electrostatic interactions and affinity toward viral envelope proteins [104]	Improved virus–surface interaction and adsorption performance [104]
UiO-66-NO_2_	Nitro-functionalized Zr-based MOF with increased surface polarity [104]	SARS-CoV-2 [104]	Surface affinity-driven adsorption and protein interaction [104]	Outstanding viral adsorption capability and enhanced virion confinement [104]
MIL-125(Ti)	Ti-based photocatalytic MOF [105]	HCoV-229E [105]	Proposed antiviral interaction following direct contact; the exact molecular mechanism remains to be elucidated [105]	79.2% reduction in viral titer after 1 h of contact [105]
HKUST-1	Cu-based MOF	HCoV-229E, SARS-CoV-2 [105]	Proposed contact-mediated viral inactivation and proposed inhibition of viral entry [105]	86% reduction for HCoV-229E and 68.4% for SARS-CoV-2 after 1 h [105]
Ag(I)-bioMOF	Three-dimensional Ag(I)-based bioMOF [106]	HAdV-36 [106]	Proposed virucidal activity following direct contact; the exact molecular mechanism remains to be elucidated [106]	>99% viral reduction reported against adenovirus [106]
ZIF-8	Zn-based MOF [107]	PEDV, BCoV, IBV, PDCoV [107]	Modulation of the ROS–mitochondria–NLRP3/STING signaling axis, enhancing host antiviral responses [107]	Broad-spectrum antiviral activity with reduced viral replication in vitro and in vivo [107]

Abbreviations: Ag(I), silver(I); Al, aluminum; BCoV, bovine coronavirus; Cu, copper; HAdV-36, human adenovirus type 36; HCoV-229E, human coronavirus 229E; HIV, human immunodeficiency virus; HKUST-1, Hong Kong University of Science and Technology-1; IBV, infectious bronchitis virus; MIL, Materials Institute Lavoisier; MOF, metal–organic framework; PEDV, porcine epidemic diarrhea virus; PDCoV, porcine deltacoronavirus; ROS, reactive oxygen species; SARS-CoV-2, severe acute respiratory syndrome coronavirus 2; STING, stimulator of interferon genes; UiO, Universitetet i Oslo; ZIF-8, zeolitic imidazolate framework-8; Zn, zinc; Zr, zirconium.

**Table 4:** Structure–function engineering strategies in hydrogel–MOF hybrid composites for regenerative and bioadsorption applications.

Hydrogel–MOF System	Structural Engineering Strategy	Key Functional Properties	Structure–Function Relationship	Potential Biomedical Relevance
Mg^2+^–gallate MOF dual-network	Dynamic Schiff-base network combined with photo-crosslinked covalent network containing embedded MOFs [113]	Enhanced mechanical strength, ROS scavenging, osteogenic activity [113]	Dual-network architecture improves injectability and structural stability, while controlled MOF degradation regulates Mg^2+^ release and biological response [113]	Bone tissue regeneration and oxidative stress modulation [113]
MOF-loaded alginate microspheres	Porous MOFs confined within magnetic alginate hydrogel beads [114]	High adsorption efficiency (~97%) and adsorption capacity (~1000 mg/g) [114]	MOF porosity provides active adsorption sites, while the hydrogel matrix regulates diffusion and structural stability [114]	Selective adsorption and bioseparation platforms [114]
ZIF-8 grown within PVA/chitosan	In situ MOF growth inside flexible polymeric matrix [115]	High flexibility, structural stability, and efficient pollutant capture under flow conditions [115]	Hierarchical pore architecture enhances mass transport, whereas polar functional groups promote interfacial adsorption [115]	Flow-through adsorption systems and biofiltration interfaces [115]
GelMA/Sr-ZIF-8	Sr-containing MOF embedded within GelMA hydrogel network [116]	Injectable, deformable, and mechanically stable bioactive scaffold [116]	Sustained Sr^2+^ release promotes regenerative signaling, while the hydrogel maintains a hydrated bioactive environment [116]	Diabetic wound healing and antimicrobial tissue regeneration [116]
Zwitterionic MOF–hydrogel coating	Zwitterionic MOFs integrated into acrylamide hydrogel coatings [117]	Selective adsorption/exclusion of biomacromolecules and antibiofouling behavior [117]	Hydrogel network regulates size-selective diffusion, whereas MOF micropores enable molecular sieving and affinity interactions [117]	Antibiofouling coatings and potential platforms for investigating enveloped virus adsorption and emerging biointerface sequestration strategies [117]

Abbreviations: MOF, metal–organic framework; ROS, reactive oxygen species; Mg^2+^, magnesium ion; PVA, poly(vinyl alcohol); GelMA, gelatin methacryloyl; ZIF-8, zeolitic imidazolate framework-8; Sr-ZIF-8, strontium-incorporated zeolitic imidazolate framework-8; Sr^2+^, strontium ion.

**Table 6:** Representative hydrogel–MOF hybrid systems exhibiting dual regenerative and antimicrobial/viral sequestration performance.

Hydrogel–MOF System	Structure–Function Mechanism	Regenerative Contribution	Antimicrobial/Antiviral Contribution
Sodium alginate- gelatin–ZIF-8	pH-responsive porous framework enabling controlled Zn^2+^ release and diffusion-mediated adsorption [155]	Promotes fibroblast proliferation, tissue remodeling, and wound closure through Zn-mediated bioactivity [155]	Zn^2+^-mediated antimicrobial activity, controlled drug release, and enhanced pathogen sequestration [155]
Enzyme–Cu-MOF	Nanozyme-like catalytic activity and ROS generation within the hydrogel matrix [156]	Supports angiogenesis and accelerates tissue regeneration under controlled oxidative conditions [156]	ROS-mediated microbial inactivation and biofilm disruption through oxidative stress mechanisms [156]
Sericin-Chitosan–Ag-MOF	Sustained Ag^+^ release combined with interfacial membrane interactions and redox activity [157]	Facilitates re-epithelialization and wound healing while maintaining cytocompatibility at optimized doses [157]	Broad-spectrum antimicrobial activity via membrane destabilization, protein denaturation, and ROS-induced nucleic acid damage [157]
Sodium alginate-polyethyleneimine–UiO-66	High-surface-area porous domains with tunable adsorption sites and controlled cargo loading [158]	Biocompatible scaffold supporting localized drug delivery and tissue repair [158]	Adsorption of bioactive molecules and delivery of antimicrobial agents with potential viral sequestration capability [158]
Stimuli-responsive hydrogel–MOF platforms	Coupled swelling–contraction behavior enabling adaptive adsorption and controlled therapeutic release (Emerging approach)	Maintains hydrated ECM-like environment and supports cellular infiltration (Emerging approach)	Dynamic pathogen adsorption and trapping and environmentally responsive antimicrobial activity (Emerging approach)
Surface-functionalized hydrogel–MOF composites	Selective interfacial interactions through electrostatic attraction and ligand-mediated recognition (Emerging approach)	Preserves tissue compatibility through localized and selective activity (Emerging approach)	Enhanced selective adsorption of enveloped viruses and inhibition of pathogen adhesion (Emerging approach)

Note: The designation “Emerging approach” refers to hydrogel–MOF design strategies currently supported primarily by proof-of-concept or early-stage experimental studies. Although these approaches have shown promising regenerative, antimicrobial, or virus–material interaction properties, direct experimental validation of their antiviral biointerface performance in wound dressing systems remains limited. Therefore, these strategies should be interpreted as prospective engineering concepts requiring further experimental and translational investigation. Abbreviations: MOF, metal–organic framework; ZIF-8, zeolitic imidazolate framework-8; UiO-66, University of Oslo-66 zirconium-based metal–organic framework; ROS, reactive oxygen species; Zn^2+^, zinc ion; Cu-MOF, copper-based metal–organic framework; Ag-MOF, silver-based metal–organic framework; Ag^+^, silver ion; ECM, extracellular matrix.

**Table 7:** Structure–function design parameters governing selective viral bioadsorption in multifunctional polymeric wound dressings.

Design Parameter	Hypothesized Mechanistic Role in Viral Bioadsorption	Potential Functional Advantages	Design Limitations/Challenges
Surface charge engineering	May promote electrostatic attraction between negatively charged viral envelopes and cationic functional domains, potentially increasing viral retention [178,179]	May enhance viral adsorption efficiency and interfacial interactions [178,179]	Excessive cationic density may induce cytotoxicity, inflammation, or local pH imbalance [178,179]
Pore architecture and microstructure	May regulate diffusion pathways, virion transport, confinement, and virus–material contact frequency, potentially increasing adsorption probability [175,176,177,180]	May improve adsorption kinetics, selective trapping, and exudate transport [175,176,177,180]	Excessively dense networks may impair oxygen diffusion and tissue infiltration [175,176,177,180]
Ligand-mediated functionalization	May enable selective recognition of viral proteins through antibodies, aptamers, or receptor-mimetic ligands [173,174,175,180,181]	May provide enhanced specificity and targeted viral sequestration [173,174,175,180,181]	Increased synthesis complexity, production cost, and ligand instability [173,174,175,180,181]
Mechanical and viscoelastic stability	May contribute to maintaining structural integrity and stable viral retention under dynamic wound conditions [171,181]	May improve durability, flexibility, and long-term dressing performance [171,181]	Mechanical degradation or structural fatigue during prolonged use [171,181]
Hydrophilic–hydrophobic balance	May modulate exudate absorption and interactions with lipid viral envelopes, potentially facilitating virus–material interactions [169,170]	May support tissue regeneration while facilitating viral destabilization [169,170]	Difficult optimization between antiviral activity and moisture retention [169,170]
MOF incorporation and interfacial interactions	May provide high-surface-area adsorption domains and active metal sites that could enhance virus–material interactions and antiviral activity [16,134,163,164,172]	May confer multifunctionality, controlled ion release, and enhanced adsorption capacity [16,134,163,164,172]	Potential particle aggregation, leaching, or reduced cytocompatibility [16,134,163,164,172]
Stimuli-responsive behavior	May enable dynamic modulation of swelling, pore size, and antiviral activity in response to pH or ROS, potentially prolonging viral confinement [160,166,167]	May support adaptive viral adsorption/sequestration and controlled therapeutic release [160,166,167]	Complex material design and response reproducibility [160,166,167]

## Data Availability

No new data were created or analyzed in this study. Data sharing is not applicable to this article.
